# Detection of sexually antagonistic transmission distortions in trio datasets

**DOI:** 10.1002/evl3.271

**Published:** 2022-01-28

**Authors:** Elise A. Lucotte, Clara Albiñana, Romain Laurent, Claude Bhérer, Genome of the Netherland Consortium, Thomas Bataillon, Bruno Toupance

**Affiliations:** ^1^ Bioinformatic Research Center Aarhus University Aarhus 8000 Denmark; ^2^ Eco‐anthropologie (EA) Muséum national d'Histoire naturelle, CNRS, Université de Paris Paris 75016 France; ^3^ Cancer Epidemiology: Gene and Environment INSERM U1018 Paris 75654 France; ^4^ Ecologie Systématique Evolution Univ. Paris‐Sud, AgroParisTech, CNRS, Université Paris‐Saclay Orsay 91400 France; ^5^ National Centre for Register‐based Research, Department of Economics and Business Economics, Aarhus BSS Aarhus University Aarhus 8210 Denmark; ^6^ Department of Human Genetics, Faculty of Medicine McGill University Montreal QC H3G 2M1 Canada

**Keywords:** Human population genetics, intralocus sexual conflict, sexual dimorphisms, sexually antagonistic selection, transmission distortion

## Abstract

Sexual dimorphisms are widespread in animals and plants, for morphological as well as physiological traits. Understanding the genetic basis of sexual dimorphism and its evolution is crucial for understanding biological differences between the sexes. Genetic variants with sex‐antagonistic effects on fitness are expected to segregate in populations at the early phases of sexual dimorphism emergence. Detecting such variants is notoriously difficult, and the few genome‐scan methods employed so far have limited power and little specificity. Here, we propose a new framework to detect a signature of sexually antagonistic (SA) selection. We rely on trio datasets where sex‐biased transmission distortions can be directly tracked from parents to offspring, and identify signals of SA transmission distortions in genomic regions. We report the genomic location of six candidate regions detected in human populations as potentially under sexually antagonist selection. We find an enrichment of genes associated with embryonic development within these regions. Last, we highlight two candidate regions for SA selection in humans.

**Impact Summary**

Sexual dimorphisms designate all biological differences between males and females, and impact both reproductive and nonreproductive traits. They arise when selection acts in opposite direction between males and females for a given trait, if this trait is encoded by the same set of genes in both sexes. This phenomenon is called an intralocus sexual conflict (IASC). Studying the evolution of sexual dimorphisms is of great interest because they impact many fundamental evolutionary processes such as sex chromosome evolution, sex determination systems, rates of divergence, and ultimately speciation. However, IASC loci are notoriously difficult to detect and previous methods developed to this end lack power and specificity. Here, we propose a new method to detect a signature of IASC using population genetic datasets structured in parents‐offspring trios: we propose to detect loci where parents transmit more often one allele to their sons and another allele to their daughters (sexually antagonistic transmission distortion). We apply this method on a human population dataset from the Netherlands and detect 66 candidate regions, including 32 containing genes that are disproportionately involved in embryonic development or expressed in developmental tissue. We further describe in details two candidate genomic regions, one located on the X chromosome and one that encompasses the Growth Hormone locus, which is involved in development and adult height. The method developed in this study can be applied to other populations and species with pedigrees genetic datasets, and will therefore help improve our understanding of IASC and sex‐dimorphisms evolution in humans and other species.

Males and females primarily differ by the size and number of their gametes, and this asymmetry generates fundamental differences in how fitness is gained in each sex (Parker et al. 1972). As a result, sexual conflict, *i.e*. when selection on a trait acts in opposite direction between the sexes, may arise. Genetic variants affecting a phenotypic trait may be favored in females but disfavored in males and vice‐versa. These traits are said to be under sexually antagonistic (SA) selection. SA genetic variations with the same genetic architecture in both sexes lead to an intralocus sexual conflict (IASC). The resolution of IASCs, notably via the evolution of sex‐biased expression, is believed to be the primary mechanism for the emergence of sexual dimorphism (Parsch and Ellegren 2013).

Although IASCs have been extensively studied, both theoretically and empirically, many fundamental questions remain unanswered (Mank 2017). In particular, their genomic signature, localization and effect on genetic diversity, is subject to debate. Theory predicts that unresolved IASCs influencing survival can lead to a stable polymorphism at SA loci (Rice 1984). It is also expected that female‐advantageous alleles are more frequently found in females than in males and vice‐versa for male‐advantageous alleles. However, a substantial difference in allelic frequency between the sexes can only occur if a large number of selective death happen in the population. In humans, although it is unlikely that such selection takes place after birth as mortality during infancy is low in Westernized populations (esa.un.org), strong selection can potentially occur before birth via spontaneous abortion. Indeed, the survival probability of an embryo is estimated to be less than 50% in humans (Benagiano et al. 2010) and differences in allelic frequencies between the sexes have been observed among human newborns (Ucisik‐Akkaya et al. 2010), suggesting that substantial amounts of sex‐biased selection may occur before birth.

Previous studies have relied on intersexual *F*_ST_ to detect ongoing IASC on survival (Cheng and Kirkpatrick 2016; Flanagan and Jones 2017; Lucotte et al. 2016; Ruzicka et al. 2021; Wright et al. 2018), but recent studies argue that this approach has limitations. Indeed, high intersexual *F*_ST_ can be observed in the absence of IASC if selection is limited to one sex or acts with different strengths in each sex. Moreover, it has low power because differences in allelic frequencies between the sexes are expected to be small and a high selection coefficient is needed for them to be detectable (Chippindale et al. 2001; Kasimatis et al. 2017, 2019).

Theory predicts that the maintenance of polymorphism at a SA locus is facilitated if linked to a distorter locus (Patten 2014; Úbeda and Haig 2005), which would lead to a transmission distortion (hereafter TD, that is, non‐Mendelian transmission of alleles to offspring) occurring before birth either at gamete production (meiotic drive), after copulation (gametic selection), or at fertilization (cryptic choice of the sperm by the ovule). Hence, haplotypes undergoing TD are expected to be enriched for variants with sex‐specific effects (Burt and Trivers 2006). Therefore, a locus undergoing IASC is likely to be transmitted in a sex‐biased way: parents would transmit more often one allele to their sons and another allele to their daughter either via selection on survival during embryonic development or via sex‐biased TD.

In this study, we propose a new approach to detect signature of IASC based on tracking the transmission patterns of alleles from parents to offspring. We rely on trio datasets and focus on sex‐biased TD in offspring. Our method models explicitly the strength and direction of TD and whether it acts in a sex‐specific manner, allowing to distinguish different types of sex‐biased TD: (i) sex‐antagonistic: one allele is preferentially transmitted to one sex and the other allele is preferentially transmitted to the other sex, (ii) sex‐differential: the same allele is preferentially transmitted with different intensities to both sexes, and (iii) sex‐limited: one of the sexes is under TD.

We first present our method. Second, we apply it on the Genome of the Netherlands (GoNL) dataset, which comprises 250 human trios sequenced at 13× coverage (The Genome of the Netherlands Consortium 2014), and explore how widespread IASCs acting on survival are in the human genome. Third, we highlight two candidate regions undergoing sex‐antagonistic TD.

## Material and Method

### DATASET AND FILTERING

The Genome of the Netherland (hereafter GoNL) dataset comprises 250 parents‐child trios (98 Sons and 150 daughters) sequenced at a median coverage of 13× (The Genome of the Netherlands Consortium 2014).

We first verified the sex labels by looking at the percentage of X chromosome heterozygosity in males (under 2%) and females (over 6%). For two couples of parents, males were mislabeled as females and vice‐versa (number 78 and 244).

Only biallelic SNPs that passed the quality control filters of GoNL were retained for further analysis (Boomsma et al. 2014; The Genome of the Netherlands Consortium 2014). The pseudoautosomal regions on the X chromosome were removed (hg19 positions, chrX:60001‐2699520 and chrX:154931044‐155260560). X‐linked SNPs presenting at least one heterozygous male were removed. Because of the trio structure of the dataset, we were able to test for Mendelian errors, and therefore marked the genotype as missing data for further analyses. Furthermore, SNPs with two or more Mendelian errors were removed. At this stage, the dataset comprised 16,980,626 SNPs genome‐wide. We removed SNPs with less than 150 informative trios (*i.e*., at least one parent in the trio is heterozygous), for autosomal loci, and 75 informative trios (*i.e*., when the mother is heterozygous) for X‐linked loci. In the final dataset, 1,709,245 autosomal SNPs and 50,204 X‐linked SNPs were kept.

We verified that the dataset was not genetically structured by sex. We calculated genetic distance matrices in parents for the autosomes and the X chromosome, independently. When computing the distances, SNPs in linkage disequilibrium (*r* ^2^ > 0.25) were removed and individuals with more than 0.5% of missing data were excluded. For autosomes, 1 million SNPs were randomly picked 10 times independently and all SNPs were included for the X chromosome. One X chromosome at random was kept for females, and this operation was iterated 30 times independently. Autosomal and X‐linked distance matrices between all individuals were calculated using the allele‐sharing distance (ASD, values between 0 and 1 [Mountain and Cavalli‐Sforza 1997]) and multidimensional scaling (MDS) was constructed from those matrices.

To test if males and females formed distinct genetic groups, using a Mantel test, we tested the correlation between two matrices: the ASD matrix between individuals and a matrix indicating for each pair of individuals whether they are of the same sex (value 0) or of different sex (value 1). Testing the correlation between those two matrices allows assessing whether within‐sex distances are different from between‐sex distances and thus whether genome‐wide data are structured by sex. For each repetition (10 random draws of 1 million SNPs for the autosomes, 30 random draws of an X chromosome in females), the correlation between both matrices was never significant, either for the autosomes or the X chromosome (see Fig. S10 for an example of one draw).

The small difference in age when sampled between males and females should not have any impact on our results neither in parents (median age of 61 years in females and 63 years in males) nor in children (median age of 35 years in females and 34 in males) (Fig. S11).

### THE LIKELIHOOD METHOD

We developed a maximum likelihood framework tailored specifically to analyze the transmission of alleles in a set of parents‐offspring trios. In our framework, all trios are assumed to be independent (genetically unrelated) and, for a given variant, we only exploit the information brought by informative trios (see Fig. 2).

Within each trio, the transmission of an allele from parents to offspring is modeled using three transmission models: *M*_0_: Mendelian transmission, *M*_1_: classical TD, and *M*_2_: sex‐specific TD (Fig. 2A). Each model makes different assumptions on the effect size and type of transmission distortion affecting a SNP. Transmission of alternative alleles is modeled via a distortion parameter (ε) that measures the strength and direction of the transmission distortion acting on the pair of alleles at a given SNP. Under Mendelian transmission (model *M*_0_), ε is equal to zero. Under classical TD (model *M*_1_), ε is different from zero but identical in both sexes. Finally, under sex‐specific TD (model *M*_2_), ε has sex‐specific values, ε_m_ for male offspring and ε_f_ for female offspring.

At each variable position where at least 150 informative trios are available (75 for the X chromosome), the natural log likelihood of the data (ln*L*) under each model is calculated as a series of binomial or multinomial probabilities. The explicit formulation of the likelihood under each model is presented in the Supporting Information. The likelihood functions under each transmission model *M*_i_ are maximized analytically thereby yielding maximum likelihood estimates for the εs as well as measures of statistical uncertainty around the ε estimates (95% approximate confidence interval from likelihood profiles). Last, likelihood ratio tests (LRTs), calculated as differences in deviance between models, are used to quantify the amount of statistical support for each alternative model. Note that all three models are nested (*M*_0_, *M*_1_, and *M*_2_) and accordingly the *P*‐values associated with each LRT statistic were calculated assuming a *χ* ^2^ probability distribution with degrees of freedom calculated as the number of fitted free parameters by which the fitted model differ: the LRT between *M*_1_ and *M*_0_ is matched against a χ^2^ distribution with 1 df, whereas *M*_2_ versus *M*_0_ is using a χ^2^ distribution with 2 df.

A local score correction method was used on the *P*‐values associated with the LRT of *M*_2_ versus *M*_0_ to account for multiple testing (Fariello et al. 2017). The local score method is designed to detect regions enriched in *P*‐values that are below a specified threshold ξ. The local scores were computed using either the recommended default setting ξ = 1 (aggregating *P*‐values < 0.1) or a more stringent threshold ξ = 2 (aggregating *P*‐values < 0.01). We computed local scores using code made available as supporting information in Fariello et al. (2017).

### CLASSIFICATION INTO SEX‐ANTAGONISTIC, SEX‐LIMITED, OR SEX‐DIFFERENTIAL TRANSMISSION DISTORTION

Each SNP with a significant *P*‐value of the LRT *M*_2_ versus *M*_0_ and located in a region enriched in low *P*‐value detected with the local score method was classified into sex‐antagonistic (SA; TD in both sexes but in different direction), sex‐limited (SL; TD in one sex), or sex‐differential (SD; TD in both sexes in the same direction but with different intensities). The decision rule was based on the value of |ε_m_ + ε_f_|, for a threshold *t* of 0.05, which corresponds to the standard deviation of the empirical distribution of the ε estimates genome‐wide.

More precisely, a SNP is classified as

- SA if |ε_m_ + ε_f_| ≤ maximum (|ε_m_|,|ε_f_|) + *t*,
- SL if |ε_m_ + ε_f_| = maximum (|ε_m_|,|ε_f_|) ± *t*, and
- SD if |ε_m_ + ε_f_| ≥ maximum (|ε_m_|,|ε_f_|) – *t*.

Then, for each region detected using the Local Score method, if at least 75% of the SNPs could be classified into one category, the region was labeled as this category, otherwise the region was labeled “mixed”.

### RECOMBINATION QUARTILE, INTERSEXUAL *F*_ST_, AND ENRICHMENT ANALYSIS IN CANDIDATE REGIONS

To investigate the relationship between presence of sex‐specific TD and recombination rate, we downloaded recombination maps from the HapMap phase II project (Frazer et al. 2007). We computed the average recombination rate for every autosomal region exhibiting a signal of sex‐specific TD. Then, we divided the genome in non‐overlapping windows matching the lengths distribution of sex‐specific TD regions, and computed the average recombination rate for these windows as a null distribution of genome‐wide recombination rates. We used binomial tests to ascertain whether the distribution of recombination rates in sex‐specific TD regions matched the null genomic distribution.

For each region type (SA, SL, and SD), the intersexual *F*_ST_ was calculated SNP‐wise using the Weir and Cockerham estimator (Weir and Cockerham 1984) and a genome‐wide distribution of *F*_ST_ was computed.

We used the RefSeq genes coordinates from built hg19 to determine which genes were located in the candidate regions. EnrichR was used to perform the functional enrichment analysis and the tissue‐expression enrichment analysis (Chen et al. 2013; Kuleshov et al. 2016).

### PIPELINE FOR ANALYSING THE CANDIDATE REGIONS

We implemented this pipeline on the regions highlighted in the main text, namely, regions chr2:229374126‐229499707, chr5:168493019‐168513628, chr7:65559180‐66028495, chr19:15838972‐15893942, and chr17:61779927‐61988014 and region chrX:47753028‐47938680. First, we phased each region using shapeit2 (O'Connell et al. 2014). A genetic distance matrix was calculated between all individuals, and an MDS was constructed. Haplotypes were identified using a density‐based clustering algorithm (package FPC, function dbscan [Hennig 2019]). Then, we determined the detailed haplotype transmission pattern and assessed significance for sex‐specific TD using a Binomial test where H0 is that the probability of transmission does not depend on offspring sex. Third, we analyzed the sequence divergence between haplotypes.

For regions chr17:61779927‐61988014 and region chrX:47753028‐47938680, we investigated the recombination landscape in TD regions. We used published sex‐specific genetic maps (Bherer et al. 2017). In total, the combined recombination dataset comprised over 3 million recombination events inferred using genome‐wide genotyping data in families and pertaining to over 100,000 meiosis. Due to sample ascertainment in the original studies, the female, male, and sex‐averaged recombination maps are mainly representative of Europeans.

## Results

### A FRAMEWORK FOR DETECTING SEX‐SPECIFIC TRANSMISSION DISTORTIONS

**Figure 1:**
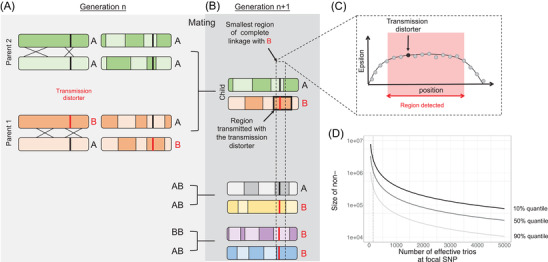
Illustration of a transmission distorter within a population. (A) One bi allelic locus (A/B), with one allele being a transmission distorter (B). In trios, we observe one meiosis event per parent. (B) One chromosome is transmitted to the child. Chromosomes carrying B are expected to be more often transmitted than A when a parent is heterozygous. The region highlighted in black is in complete linkage with the transmission distorter as no recombination occurred within this region across trios. As we consider more trios, and more events of recombination, the region in complete linkage with B gets smaller and we gain resolution on the exact position of the transmission distorter. (C) Schematics of the region detected compared to the position of the transmission distorter. The size of the region detected with a high ε is proportional to the number of trios. (D). The expected size of nonrecombining window, that is, the smallest region of complete linkage with B, in relation to the number of informative trios at the focal SNP, considering high, medium, and low recombination. Assuming a recombination rate of 1cM/Mb, the number of recombination events in 1Mb is Poisson distributed (with mean λ = 0.01), therefore the first recombination breakpoint downstream of the focal SNP is exponentially distributed with mean 1/ λ. The closest position among the *N_T_* trios is also exponential with mean 1/(*N_T_* λ). The same reasoning applies to the upstream recombination breakpoint. Because the positions of the upstream and downstream recombination breakpoints are independent and identically distributed, the length of the recombination‐free region is gamma‐distributed with a shape parameter of 2 and a rate parameter of (*N_T_* λ) (quantiles of the distribution are represented in panel D).

We developed a likelihood‐based framework to detect sex‐biased TD in offspring using trio sequencing (or genotyping) datasets. Using a trio dataset, we detect transmission distortion over one generation, and only one meiosis per individual (Fig. 1A). SNPs physically close to a transmission distorter are transmitted along within a recombination‐free block (Fig. 1B, C). With a small sample size, we can only detect large physical regions containing a distorter with a strong effect. As more trios are available, more resolution is gained on the exact position of the transmission distorter, as more events of recombination will render SNPs transmission gradually more independent (Fig. 1D).

**Figure 2:**
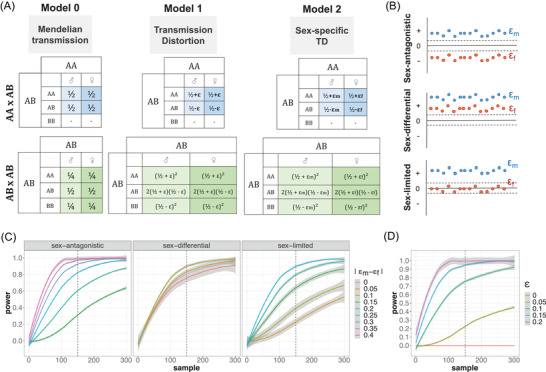
Overview of the likelihood method. (A) Probability of transmission tables for each model, for AA×AB parents and AB×AB parents. Model 0 is Mendelian transmission, Model 1 is standard transmission distortion, with a unique distortion parameter ε, and Model 2 is sex‐specific transmission distortion with sex‐specific distortion parameters: ε_m_ for males and ε_f_ for females. (B) Schematics of the inferred sex‐specific distortion parameters in sex‐antagonistic, sex‐differential, or sex‐limited regions. (C) Power simulations at a 0.05 significance level to detect sex‐specific TD in case of sex‐antagonistic, sex‐differential, or sex‐limited TD, as a function of the number of informative trios and depending on the absolute value of the differences between ε_m_ and ε_f_. (D) Power simulation at a 0.05 significance level to detect different value of a non‐sex‐specific ε for different values of ε and number of informative trios.

This likelihood‐based method is applied throughout the genome at informative biallelic SNP, that is, SNP with at least one heterozygous parent. At each focal SNP, patterns of transmission from parent to offspring are fitted under three alternative models (Fig. 2A). All models incorporate a distortion parameter (ε) that measures the strength and direction of the transmission distortion acting on the pair of alleles at a given SNP: ε is zero under Mendelian transmission (model *M*_0_), different from zero but identical in both sexes under classical TD (model *M*_1_), and ε has sex‐specific values (ε_m_ for male offspring and ε_f_ for female offspring) in case of sex‐specific TD (Model *M*_2_). An LRT is performed between *M*_0_ and *M*_2_ to detect loci under sex‐biased TD.

The sex‐specific ε parameter estimates are then used to classify a SNPs exhibiting a sex‐biased TD signal into sex‐antagonistic (SA: TD in both sexes and in opposite direction), sex‐limited (SL: TD in only one sex), and sex‐differential (SD: TD in both sexes and in the same direction but with different strengths) (see *Methods* and Fig. 2B). Genomic regions with 75% or more SNPs with one type of TD signal were categorized as such, and those that could not be consistently classified were labeled “mixed” regions.

We performed power analyses on simulated trio data to evaluate the ability of our method to detect SNPs that undergo sex‐biased TD (Fig. 2C, D). We implemented two types of simulations, one with equal transmission to male and female offspring and one with sex‐specific distorsion parameters; each scenario was simulated with 500 repetitions. We varied the number of informative trios available, the difference between ε_m_ and ε_f_ from 0 to 0.4 (|ε_m_ – ε_f_|; Fig. 2C), and the magnitude of the ε affecting a SNP from 0 to 0.2 (Fig. 2D). The power corresponds to the proportion of significant *P*‐values accross repetitions (using type‐I error α = 0.05). As expected, power increases with the sample size and the effect size. We find that the power to detect SA is strongly influenced by the sample size of the trio dataset (Fig. 2). For sex‐antagonistic TD, and with a sample of 150 informative trios, the power is 0.6 for |ε_m_ – ε_f_| = 0.2, 0.8 for |ε_m_ – ε_f_| = 0.25, and 0.95 for |ε_m_ – ε_f_| = 0.3 (Fig. 2C). Moreover, for 150 informative trios, we have a power of 0.75 to detect an ε of 0.1, of 0.9 for an ε of 0.15, and 1 for an ε of 0.2 (Fig. 2D). The cutoff of 150 informative trios we used in this study provides sufficient power to detect sex‐biased TD within the sample size of the GoNL trio dataset.

The LRT(*M*_0_‐*M*_2_) *P*‐values we obtained for individual SNPs can be analyzed further using method controlling false discovery rate or a local score method (Fariello et al. 2017). By doing the latter, we focus on regions enriched in low *P*‐values, mitigating the common issue of the “winner's curse” in genome‐scan approaches and accounting for the fact that several loci that are physically close to a target of TD share the same signal due to linkage disequilibrium.

Our method can be used for both NGS and array‐based genotyping datasets, keeping in mind that regions poorly represented in a SNP genotyping chip will be less likely to be considered significant by the local score method. Below we illustrate our method by applying it to the sequencing GoNL trios dataset (The Genome of the Netherlands Consortium 2014).

### GENOMIC DISTRIBUTION OF SEX‐BIASED TRANSMISSION DISTORSION

**Figure 3:**
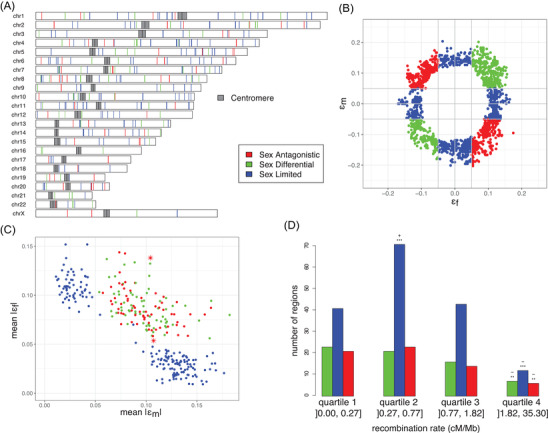
Description of the Sex Antagonistic (Red), Sex Differential (green), and Sex Limited (blue) signal. (A) Genomic localization of the TD regions. The dark gray rectangles represent centromeres. (B) Classification of the ε‐ example of chromosome 1. Each point represents one SNP, for which an epsilon for males (ε_m_) and epsilon for females (ε_f_) were estimated. (C) Classification of the regions detected using the Local score method. Each point represents a region, for which a mean value of the absolute ε_m_ and ε_f_ was calculated. The two stars highlight the two regions we analyze further. (D) Number of regions per quartile of recombination rate (cM/Mb). Each bar represents one type of region. Stars represent the level of significance as measured by a binomial test (H0: 25% of the regions are in each quartile of recombination rate, **P‐value < 0.1, ***P‐value < 0.01). The sign (+ or –) indicates an excess or a deficit of regions as compared to H0.

We analyzed 248 trios from GoNL sequenced at a median coverage of 13×. After filtering for informative trios, we screened a total of 1,709,245 SNPs on autosomes and 50,204 X‐linked SNPs. We performed an LRT between model M_0_ (no TD) and model M_2_ (sex‐specific TD) for each of these SNPs and estimated the sex‐specific ε. We applied the Local Score method to detect regions that are enriched in low *P*‐values for the LRT, using a threshold of ξ = 1, which considers *P*‐values lower than 0.1. Regions detected using a threshold of ξ = 2, which considers *P*‐values lower than 0.01, are listed in Table S1. With the threshold ξ = 1, we detected 66 SA candidate regions in the GoNL data, including 32 containing genes. Moreover, we detected 168 SL regions, 68 SD regions, and 230 mixed regions (Fig. 3A; Tables 1 and S1). The ε values for single SNPs classified as SA, SD, and SL are displayed on Figure 3B for chromosome 1 as an example. Figure 3C shows the mean absolute values of ε_m_ and ε_f_ for the regions detected as enriched in low *P*‐values using the local score method.

**Table 1:** Summary table describing the regions where sex‐specific TD was detected

				Number of SNPs per Region		
Type	Number of Regions	With Genes	Median Length (bp)	Median	Min	Max
Sex antagonist	66	32	72,005	66	14	374
Sex limited	168	70	68,578.5	77	13	720
Sex differential	68	33	74,368	60.5	18	238
Mixed	230	121	98,567	92.5	13	1111

We examined the robustness of our findings. First, if we use a more stringent cutoff to aggregate local score (i.e., ξ = 2), we find 38 regions: 11 SA, 12 SD, 7 SL, and 8 mixed regions (Table S1). As an alternative, we computed an estimate of the proportion of regions that are best explained by model *M*_0_ that posits “no transmission bias” (proportion π_0_) versus the proportion of region exhibiting some form of distortion (1 – π_0_). To do so, we first conservatively thinned out SNPs, keeping only SNPs that are 500kb apart to minimize correlation among *P*‐values (*n* = 5272). We obtain a very uniform empirical distribution of *P*‐values across these SNPs, as expected if our LRT is well calibrated and most tests in thinned SNPs are anchored in regions coming from *M*_0_ (Fig. S1). Using the empirical distribution of thinned *P*‐values, a false discovery rate approach (*q*‐value) estimates that, depending on the cutoff used to estimate π_0_, π_0_ is 0.98‐0.99. This is suggesting that 1 – π_0_ ≈ 1%‐2% of the SNPs are anchored in regions that harbor a signal of transmission distortion. This corresponds to roughly 50‐10 regions departing from *M*_0_. Note, however, that when assuming a strict FDR approach only one SNP yields a signal that is strong enough to have local FDR < 0.01. This illustrates that more trios are needed to get more precision on the π_0_ estimate (as more data will generate clearer separation between the distributions of *P*‐values associated to SNPs coming from either *M*_0_ or alternative SA models such as *M*_2_).

Finally, we investigated the distribution of TD regions with respect to recombination. SA, SL, and SD regions are significantly underrepresented in the high‐recombination quartile of the genome (Fig. 3D; *P*‐value = 4.41 × 10^−3^, 3.23 × 10^−9^, and 2.27 × 10^−3^ for SD, SL, and SA regions, respectively); however, this could be due to the Local Score method used to detect regions enriched in low *P*‐values. Indeed, a high recombination rate implies a low LD, which in turns leads to less power to detect significant regions using the Local Score method. SL regions are significantly overrepresented in region of medium‐low recombination (quartile 2, ]0.27, 0.77] cM/Mb, *P*‐value = 9.56 × 10^−7^).

### INTERSEXUAL *F*_ST_ DISTRIBUTIONS FOR THE THREE TYPES OF REGIONS

For each type of regions (SA, SL, and SD), we computed the distribution of intersexual *F*_ST_ in offspring, and compared it to a matched empirical null distribution of intersexual *F*_ST_. This null distribution was obtained by randomly sampling genomic regions with matching nucleotide diversity, length, and number of SNPs (Fig. S2). SA, SL, and SD regions show higher values of intersexual *F*_ST_, as compared to matched regions. Among TD regions, the values for SA regions are significantly higher than both SL and SD (Wilcoxon‐Mann‐Whitney test, *P*‐values < 2 × 10^−16^). Indeed, for SA, SD, and SL regions, the means for the intersexual *F*_ST_ values in offspring are 0.012 (SD = 0.011), 0.000 (SD = 0.004), and 0.005 (SD = 0.009), respectively. This result is consistent with the expectation that intersexual *F*_ST_ should be high in regions harboring signals of IASC on survival and that high values can also be detected in case of SL and SD selection (Mank 2017; Wright et al. 2018).

Next, we used the 1000 Genomes European populations to test if our candidate regions harbored footprints expected if SA variants are also under selection in other populations. We find that intersexual *F*_ST_ significantly decreases with distance to the center of the strongest candidate SA regions (ξ = 2; Supporting Information III.1, linear regression beta = –1.81 × 10^−4^, *R* ^2^ = 3.77 × 10^−3^, *P*‐value = 9.00 × 10^−5^), as expected from theory (Kirkpatrick and Guerrero 2014), whereas it is not the case for SD and SL regions (beta = –7.37 × 10^−5^, *R* ^2^ = 4.13 × 10^−4^, *P*‐value = 0.151 and beta = 8.69 × 10^−5^, *R* ^2^ = 1.15 × 10^−3^, *P*‐value = 0.120, respectively).

### FUNCTIONAL ENRICHMENT ANALYSIS

We performed a functional enrichment analysis, focusing on the gene ontologies for biological process and a tissue expression enrichment (Human gene Atlas, GTEx, and Jensen tissue, see *Methods*) within the list of genes located in SA, SL, and SD regions, using EnrichR (Chen et al. 2013; Kuleshov et al. 2016). Interestingly, genes present in SA regions are enriched in genes associated with embryonic development (Table 2), both functionally (the growth hormone receptor signaling pathway) and for tissue expression (developmental tissues, for example, placenta, umbilical artery, amniotic fluid). The genes contributing most to enrichment signals are the growth hormone genes (GH2, CSH1, CSHL1, CSH2), which are located in a cluster on chromosome 17, that we will henceforth refer to as the GH locus. Additionally, we find that there is an enrichment in genes that are downregulated in the uterus and upregulated in the adipose tissue in females. Genes located in SD and SL regions do not show enrichment in sex‐specific functions or development (Tables S2 and S3). However, genes located in the mixed regions are expressed preferentially in sex‐specific tissues or are enriched in functions related to embryonic development: genes downregulated in the fallopian tubes and genes involved in embryonic lethality between implantation and somite formation (Table S4).

**Table 2:** Summary of the enrichment found for the list of genes present in SA TD regions. For GO Biological Process and Human Gene Atlas, the enrichment is based on biological functions. For both GTEX and Jensen Tissue databases, the enrichment is based on which tissues the genes are expressed in

Database	Term	Overlap	*P*‐value	Adjusted *P*‐value	*Z*‐score	Combined Score	Genes
GO Biological Process	Growth hormone receptor signaling pathway (GO:0060396)	3/21	1.03 × 10^–4^	5.65 × 10^–2^	–2.15	19.77	GH2; CSH1; CSHL1
Human Gene Atlas	Placenta	5/405	3.32 × 10^–2^	9.79 × 10^–1^	–1.66	5.65	GH2; CSH2; CSH1; OLR1; CSHL1
GTEX Tissue Expression Profile down	GTEX‐QCQG‐1326‐SM‐48U24_uterus_female_50‐59_years	4/261	2.82 × 10^–2^	1.00	–1.93	6.88	EPHA10; PPP1R1C; SOD2; ADRA1A
GTEX Tissue Expression Profile up	GTEX‐OXRO‐0226‐SM‐3LK6F_adipose tissue_female_60‐69_years	11/1039	5.79 × 10^–3^	1.00	–1.84	9.48	BIN3; WTAP; SLC6A16; STRADA; NTRK3; TIMM9; TCP1; DDX42; SOD2; SORBS3; CCAR2
Jensen Tissues	Umbilical_artery	2/8	5.27 × 10^–4^	8.04 × 10^–2^	–2.83	21.39	CSH2; CSH1
	Endometrial_gland	2/10	8.42 × 10^–4^	8.04 × 10^–2^	–3.55	25.15	CSH2; CSH1
	Trophoblast_cell_line	2/11	1.03 × 10^–3^	8.04 × 10^–2^	–2.99	20.61	CSH2; CSH1
	Amniotic_fluid	3/52	1.56 × 10^–3^	9.15 × 10^–2^	–3.14	20.31	CSH2; CSH1; THY1

### CASE STUDY OF TWO POTENTIAL SEXUALLY‐ANTAGONISTIC REGIONS

We identified 66 SA regions, including 32 containing genes (Table S1). We selected six of those regions, representing both tails of the distribution of mean delta |ε_m_ – ε_f_|, mean ε_m_, mean ε_f_, and number of genes, plus the only X‐linked region (Table S1). Region chr2:229374126‐229499707 has the highest mean ε_f_, region chr5:168493019‐168513628 has the highest delta |ε_m_ – ε_f_| and the highest mean ε_m_, region chr7:65559180‐66028495 has the lowest delta |ε_m_ – ε_f_|, chr17:1761779927‐61988014 has the lowest mean ε_f_ and the most genes, chr19:15838972‐15893942 has the lowest mean ε_m_, and chrX:47753028‐47938680 is the only SA region on the X chromosome. We chose to present in details only two regions: the region chr17:1761779927‐61988014, which has the highest number of genes and is therefore more likely to have a functional impact, and the region on the X chromosome (chrX:47753028‐47938680), an expected hotspot for the accumulation of SA loci (Lucotte et al. 2016; Rice 1984).

#### Region on chromosome 17 (chr17:61779927‐61988014, 208kb)

**Figure 4:**
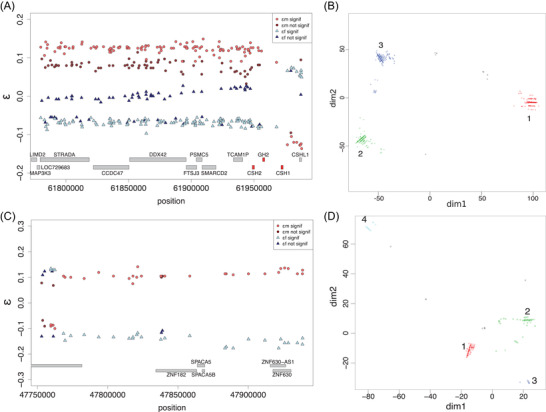
Sexually antagonistic TD candidate regions (A, B) chr17:61779927‐61988014 (208 kb) and (C, D) chrX:47753028‐47938680 (186 kb). (A, C) Sex‐specific distortion coefficients ε (ε_m_ in red and ε_f_ in blue). ε differing significantly from zero (LRT *P*‐values lower than 0.05) are in lighter colors. (B, D) Multi‐dimensional scaling on the genetic distance matrix between all haplotypes (two per individual) based on the phased data, clustered using a density‐based clustering algorithm. Each color denotes a cluster. For A, note that the inversion of the signs of the ε in the vicinity of CSHL1 gene is due to a different attribution of reference and alternative alleles and does not affect our pipeline to detect SA regions.

This region contains part of the GH locus: CSH1, CSH2, GH2, and CSHL1 (GH1 missing), which are responsible for most of the functional and tissue‐expression enrichment in genes located in SA regions (Fig. 4A, B; Table 2).

The absolute values of the ε are comprised between 0.032 and 0.149 (mean 0.107) for males and 0.00 and 0.097 (mean 0.054) for females, with a mean delta |ε_m_ – ε_f_ | of 0.158 (Fig. 4A). High intersexual *F*_ST_ values are observed in this 208kb region, both in children and parents (Fig. S3A). However, SNPs located next to each other can harbor low and high intersexual *F*_ST_, suggesting a complex genomic architecture. We phased this region and discovered three distinct haplotypes (Figs. 4B and S4). The three haplotypes are almost equally distributed in children (1: 35.8%, 2: 33.8%, 3: 28.2%) and in parents (1: 35.1%, 2: 29.1%, 3: 34.1%). In parents, haplotype 2 is carried by fewer males than expected at random (41.38% males, p_0_ = 50.20%, Binomial test *P*‐value = 0.002), whereas haplotype 3 is carried by more males than expected at random (57.35% males, p_0_ = 50.20%, Binomial test *P*‐value = 0.01). In children, we found an excess of male carrying haplotype 1 (48.04% males, p_0_ = 39.20%, Binomial test *P*‐value = 0.02), whereas haplotype 2 is still carried by fewer males than females, although not significantly (31.95% males, p_0_ = 39.20%, Binomial test *P*‐value = 0.06). Transmissions of these three haplotypes seem to be sex‐antagonistic (Table S5): if a parent is heterozygous for haplotype 1 and 3, haplotype 1 is more often transmitted to sons (Fisher exact test, *P*‐value = 3.5 7 × 10^−2^) and haplotype 3 to daughters (Fisher exact test, *P*‐value = 4.79 × 10^−2^). Additionally, if the heterozygous parent has haplotype 1 and haplotype 2 or 3, haplotype 1 is more often transmitted to sons (Fisher exact test, *P*‐value = 3.84 × 10^−3^) and haplotype 2 or 3 to daughters (Fisher exact test, *P*‐value = 4.48 × 10^−2^). The sample sizes are small, and these *P*‐values do not resist correction for multiple testing, except for the biased transmission of haplotype 1 to sons compared to 2 or 3 (*P*‐value = 3.84 × 10^−2^ after Bonferroni correction). These results suggest that haplotype 1 is beneficial for males and deleterious for females.

This region encompasses 1291 SNPs, and has a length of 208,087bp. The mean number of differences between genomics regions is 167.66 SNPs for haplotypes 1 and 2, 151.78 SNPs for haplotypes 1 and 3, and 84.59 SNPs for haplotypes 2 and 3 (Table S6). For such a short region, this suggests that recombination is rare between the three haplotypes. Indeed, this region is a cold‐spot of recombination (Fig. S3B), flanked by two sex‐specific hotspots of recombination.

This pattern is not due to a mapping artifact, either sex specific or region specific (Supporting Information II). Although we could not replicate the transmission results in another dataset because we do not have access to a dataset with enough trios, we were able to replicate the finding of the three haplotypes in other European populations (Supporting Information III.2). However, we did not find a pattern of preferential association between sex and any of the three haplotypes in other datasets (Supporting Information III.2).

#### Region on the X chromosome (chrX:47753028‐47938680, 186kb)

This region contains the gene SPACA5, the only gene from the SPACA family (five genes) located on the X chromosome (Fig. 4C, D). It encodes a sperm acrosome‐associated protein, which is directly involved in gamete fusion.

The absolute values of ε are comprised between 0.068 and 0.141 (mean = 0.104) for males and 0.108 and 0.177 for females (mean = 0.138) and the mean |ε_m_ – ε_f_| is 0.242 (Fig. 4C). High intersexual *F*_ST_ can be observed along the whole region in offspring, and at the right end of the region for parents (Fig. S3C). The same pattern of alternating high and low *F*_ST_ for adjacent SNPs, similar to the region of chromosome 17 highlighted above, was observed. We discovered four haplotypes, including two in high frequency: haplotype 1 and haplotype 2 with a frequency of 0.516 and 0.441 in parents (0.528 and 0.428 in children), respectively (Figs. 4D and S5). Haplotype 3 and 4 have a frequency of 0.013 and 0.025 in parents (0.012 and 0.026 in children), respectively. Transmission of these haplotypes is significantly sex biased (Table S7): haplotype 1 is more often transmitted to daughters (Fisher exact test *P*‐value = 2.95 × 10^−2^), whereas haplotype 2 is more often transmitted to sons (Fisher exact test *P*‐value = 2.05 × 10^−2^). Interestingly, when fathers have haplotype 1, mothers are more likely to transmit haplotype 1 to daughters (24 cases against 10 cases of mothers transmitting haplotype 2, Binomial test *P*‐value = 2.43 × 10^−2^). Haplotypes 1 and 2 have a lower percentage of divergence in sequence than what we observe for the region on chromosome 17 (Table S8), which can be explained by the occurrence of recombination within the region (Fig. S3D).

As above, validation analyses suggest that this pattern is not due to a mapping artifact (Supporting Information II). European populations from the 1000 Genomes project display a similar haplotype structure (Supporting Information III.2), but no enrichment of males or females bearing one haplotype (Supporting Information III.2).

#### Other regions

Results from the other four regions (chr2:229374126‐229499707, chr5:168493019‐168513628, chr7:65559180‐66028495, and chr19:15838972‐15893942) are presented in Figures S6‐S9 and Tables S9‐S10. We also discover several haplotypes (Figs. S6‐S9) that are transmitted in a sex‐specific manner (Table S9), except for the chr7:65559180‐66028495 region that does not show significant sex‐specific distortion (type‐I error α = 0.05) at the haplotype level. The haplotypes show high differentiation (Table S10), the maximum pairwise distances for each region being 19.48%, 16.76%, 13.48%, and 24.92%, respectively.

## Discussion

We propose a new method to detect sex‐of‐offspring‐specific TD, hereafter referred to as sex‐biased TD, using sequencing or genotyping of trio (parent‐offspring) datasets to track directly the transmission of alleles in each sex. This offers a way to categorize different types of genomic regions: Sex‐Antagonistic (SA), Sex‐Limited (SL), and Sex‐Differential (SD) TD by providing estimates of the intensity of sex‐specific TD. This method circumvents the limitation of previous methods relying solely on intersexual *F*_ST_, by specifically detecting loci undergoing SA TD. Moreover, by using a Local Score method (Fariello et al. 2017) we detect genomic windows with an enrichment in low *P*‐values, as expected under TD, and hence reduce the risk to detect single SNPs with artefactual high *F*_ST_ as compared to single‐locus *F*_ST_ approaches.

Loci undergoing IASC are expected to experience balancing selection, because different alleles are beneficial in different sexes (Connallon and Clark 2014). It has been proposed to use Tajima's *D*, a statistic summarizing the site frequency spectrum (essentially capturing the amount of rare vs. frequent alleles), in combination with intersexual *F*_ST_ to distinguish SA selection from other sex‐biased selections (Wright et al. 2018). However, signatures of balancing selection are notoriously difficult to detect (Rowe et al. 2018). Moreover, in our case, because we only keep SNPs with at least 150 heterozygous trios (75 for the X chromosome), we have an ascertainment bias toward SNPs with an elevated Tajima's *D*, whether they show a signal of TD or not.

The power of the method to detect regions with distortions is strongly dependent on the number of informative trios. Because we have 248 trios in GoNL, we have statistical power to only test SNPs with intermediate frequencies, that is, which segregate in enough informative trios to detect a transmission distortion. We expect SNPs directly under SA selection to be at intermediate frequencies (Mank 2017) and to exhibit a large difference in sex‐specific distortion parameter, as measured by (|ε_m_ – ε_f_|), so SNPs with the strongest amount of SA TD are specifically captured by our method. Although GoNL is one of the largest trio datasets published to date, the limited number of trios precludes from over‐speculating on individual regions. In this study, we draw conclusions on overall patterns of SA TD, and merely focus on two regions that seemed the most striking and interesting examples of SA TD.

We detected 66 SA TD regions genome‐wide, including 32 with genes. We found that regions undergoing SA TD are enriched for SNPs with high intersexual *F*_ST_, which is expected (Lucotte et al. 2016; Mank 2017). Regions undergoing SL and SD TD also show high intersexual *F*_ST_, but have significantly lower intersexual *F*_ST_ than SA TD regions.

We performed an enrichment analysis on the list of genes located within the SA regions to determine if these genes are significantly involved in a specific function or expressed in a specific tissue. The enrichment analyses performed in SA regions reveal that they contain genes that are primarily involved in developmental functions, and expressed in tissues involved in development. The functional enrichment and expression enrichment were not significant after correction for multiple testing. However, this result is in concordance with the expectation that SA TD may occur during gamete fusion and embryo development.

We then focused on six SA TD regions representing both extremes of the distribution of mean delta |ε_m_ – ε_f_|, mean ε_m_, mean ε_f_, and number of genes. For all but one investigated regions, we detected several haplotypes that are preferentially transmitted to one sex or the other, which is in concordance with the prediction of theoretical works (Burt and Trivers 2006; Patten 2014; Patten et al. 2010; Ubeda et al. 2011; Úbeda and Haig 2005). The haplotypes display high pairwise sequence divergence. We investigated in more details two regions: a region on chromosome 17 containing the genes responsible for most of the genome‐wide enrichment in developmental tissues and the unique SA region containing genes detected on the X chromosome.

The chromosome 17 region encompasses the growth hormone locus, notably the GH gene, which encodes a protein in the placenta that is important for *in utero* development (Oberbauer 2015), and affects adult traits such as height and bone mineral density (Timasheva et al. 2013). Interestingly, there is evidence for ongoing IASC on human height (Stulp et al. 2012). The high sequence divergence among the three haplotypes is probably due to the lack of recombination in this region. Although sample sizes are low, a pattern of SA TD of the haplotypes can be detected. However, the *P*‐values for sex differences in haplotype transmission are nonsignificant after correction for multiple testing.

The X chromosome region encompasses the only SPACA gene on this chromosome, which is expressed in the spermatozoid acrosome, involved in gamete fusion. This is an interesting feature as TD could happen at gamete fusion. Deeper investigations of the role of this gene and the impact of the observed genetic polymorphism are warranted.

We were able to replicate the finding of the number of haplotypes in European populations of the 1000 Genomes dataset for those two regions, but not the sex‐specific enrichment within the haplotypes. However, we found that the intersexual *F*_ST_ significantly decreases with distance to the center of the strongest candidate regions (ξ = 2) in 1000 Genomes European populations, which suggests that SA selection is also ongoing in these genomic regions within those populations. A trio dataset of at least equal sample size should be investigated in the future to validate the TD pattern detected in the GoNL data. In the near future, we expect more datasets with pedigrees (trios or extended sib ships), on which this method could be used to gain more knowledge on the architecture of SA TD in the human genome.

TD can be due to several nonexclusive mechanisms: haploid selection during gamete formation, selection occurring between gamete formation and fertilization, SA selection occurring between fertilization and birth (during embryonic development), and after‐birth SA selection on survival. Our method does not allow to distinguish between these biological mechanisms. One perspective of this study would be to modify the method to consider the sex of the parent in TD, which could allow to distinguish between TD occurring before and after fertilization. Indeed, variation in expression profile of the genes in haploid sperm among a single ejaculate has been shown to correlate with motility and fertility in humans, which is consistent with gametic selection happening in humans (Lambard et al. 2004).

## Conclusion

We provide a new framework to detect loci specifically undergoing sex‐antagonistic TD in genomic datasets. It allows to discriminate between sex‐antagonistic, sex‐limited, and sex‐differential TD. This circumvents limitations of the intersexual *F*_ST_ used in previous studies. We detect 32 gene coding regions undergoing sex‐antagonistic TD in a human population from the Netherlands and highlight two intriguing candidate regions. Our method can be applied to any sequencing or genotyping datasets structured in parents‐offspring trios, and constitutes therefore an important progress to elucidate the genomic architecture of IASCs and their implications in sexual dimorphisms evolution. As costs of sequencing and genotyping are rapidly decreasing, we expect pedigrees datasets to become commonplace in the future.

## AUTHOR CONTRIBUTIONS

EL, BT, and TB conceived and designed the study and acquired funding. This work was supervised by TB and BT. TB and BT formalized and CA coded the likelihood method designed by TB and EL. EL and RL curated and analyzed the data. Additional analyses on recombination were performed by CB. EL drafted the initial version of the manuscript and BT, RL, TB, and CA contributed to later versions of the manuscript. The Genome of the Netherland Consortium provided the data.

## DATA ARCHIVING

This project was approved by the GoNL Data Access Committee (application no. 2014053).

Associate Editor: Z. Gompert

## Supporting information

SUPPLEMENTARY TEXT

Supplementary Table S1
