# Supplementary material for: Detection of sexually antagonistic transmission distortions in trio datasets
Source: Evol Lett. 2022 Jan 28;6(2):203–16. doi: 10.1002/evl3.271 (PMC8966469; doi:10.1002/evl3.271)
Supplement: Supplementary file 1 — SUPPLEMENTARY TEXT [file EVL3-6-203-s002.pdf]

SUPPLEMENTARY TEXT

Complete list of authors from Genome of the Netherland Consortium:

Dorret I Boomsma, Cisca Wijmenga, Eline P Slagboom, Morris A Swertz, Lennart C Karssen, Abdel Abdellaoui, Kai Ye, Victor Guryev, Martijn Vermaat, Freerk van Dijk, Laurent C Francioli, Jouke Jan Hottenga, Jeroen F J Laros, Qibin Li, Yingrui Li, Hongzhi Cao, Ruoyan Chen, Yuanping Du, Ning Li, Sujie Cao, Jessica van Setten, Androniki Menelaou, Sara L Pulit, Jayne Y Hehir-Kwa, Marian Beekman, Clara C Elbers, Heorhiy Byelas, Anton J M de Craen, Patrick Deelen, Martijn Dijkstra, Johan T den Dunnen, Peter de Knijff, Jeanine Houwing-Duistermaat, Vyacheslav Koval, Karol Estrada, Albert Hofman, Alexandros Kanterakis, David van Enckevort, Hailiang Mai, Mathijs Kattenberg, Elisabeth M van Leeuwen, Pieter B T Neerincx, Ben Oostra, Fernanodo Rivadeneira, Eka H D Suchiman, Andre G Uitterlinden, Gonneke Willemsen, Bruce H Wolffenbuttel, Jun Wang, Paul I W de Bakker, Gert-Jan van Ommen & Cornelia M van Duijn

I- **The likelihood method- Derivation of the mathematical formulas**

II- **Is the sex-specific pattern of transmission due to a mis-mapping of the reads?**

1. **BLAST analysis**
2. **Analyses of the coverage**

III- **Replication in the 1000Genomes populations**

II- Is the sex-specific pattern of transmission due to a mis-mapping of the reads?

1- BLAST analysis

For both regions included in the case study, we investigated whether peculiar genomic properties could explain the TD signal we detect. More specifically, sequence similarity between a given region and a sex chromosome could result in mapping errors, eventually leading to the detection of a spurious sex-biased TD signal. To avoid this caveat, we performed a blast analysis as follows. We downloaded the GRCh37 reference genome from the Ensembl database [1]. Then, for each region of interest, we extracted the corresponding sequence and divided it in 10,000 random fragments of 100 bp. We blasted these fragments against the human genome and recorded hits outside the region of interest with at least 95% identity into a set of hits. Finally, we computed the number of hits from this set on each chromosome, and used a binomial test to test whether the number of hits on the sex chromosomes were different than expected by chance, taking into account chromosome lengths. Results are given in Table II.1. For both region, the number of hits is significantly lower than expected at random, given the length of the region.

Table II.1: Results of the blast analyses

Query	target	# hits observed	# hits expected	pvalue
chr17, 95% identity	X	424,632	496,525.8	4.44E-323
chr17, 95% identity	Y	34,489	188,779.7	2.96E-323
chrX, 95% identity	Y	66,004	179,674.3	2.96E-323

2- Analyses of the coverage

Chromosome 17 region chr17:61779927-61988014

We extracted all positions from chromosome 17 with a filter == PASS. For each position, we computed the mean of the coverage for all males and all females separately. Then we calculated the mean over all positions per bin of 1,000 bp. Within the whole chromosome bins, we filtered bins with a mean coverage outside of the minimum and maximum coverage of the region of interest, to avoid outliers due to specific regions. The mean coverage over 1000bp bins for the region of interest is presented on figure II.1A. The mean coverage is very close for males and females (13.90 and 13.50, respectively) for the region of interest and the whole chromosome (13.93 and 13.55, respectively, figure II.1B).

Then, for each sex, we randomly draw 1000 times the number of bins in the region of interest bins from the genome-wide bins (208 bins) and we calculated the difference between the mean coverage of this random sample of bins and the region of interest. None of the comparison yielded a difference of 1 read or more (figure II.1C). In conclusion, there is no artefact due to coverage on the region of interest.

X chromosome region chrX:47753028-47938680

We extracted all X-linked position with a filter == PASS, outside the pseudo-autosomal regions.

The same analyses as above was perform. The mean coverage over 1000bp bins for the region of interest is presented on figure II.2A For males, the mean coverage is 7.50 (7.63 genome-wide, figure II.2B) and for female it is 14.55 (14.71 genome-wide, figure II.2B).

Then, for each sex, we randomly draw 1000 times the number of bins in the region of interest bins from the genome-wide bins (171 bins), and we calculated the difference between the mean coverage of this random sample of bins and the region of interest. None of the comparison yielded a difference of 1 read or more (figure II.2C). In conclusion, there is no artefact due to coverage on the region of interest.

Figure II.1- Coverage analyses for the chromosome 17 region. A- Mean coverage for males (red) and females (blue), the ribbon corresponds to the mean +- sd, for the region of interest. **B-** Distribution of the mean coverage for females and males for the whole chromosome 17 (top panels) and for the region of interest for females and males (bottom panel). **C-** Distribution of the difference between the mean coverage of the region of interest and the mean coverage of a randomly drawn set of 208 bins in the whole chromosome 17, for 1000 random samplings.

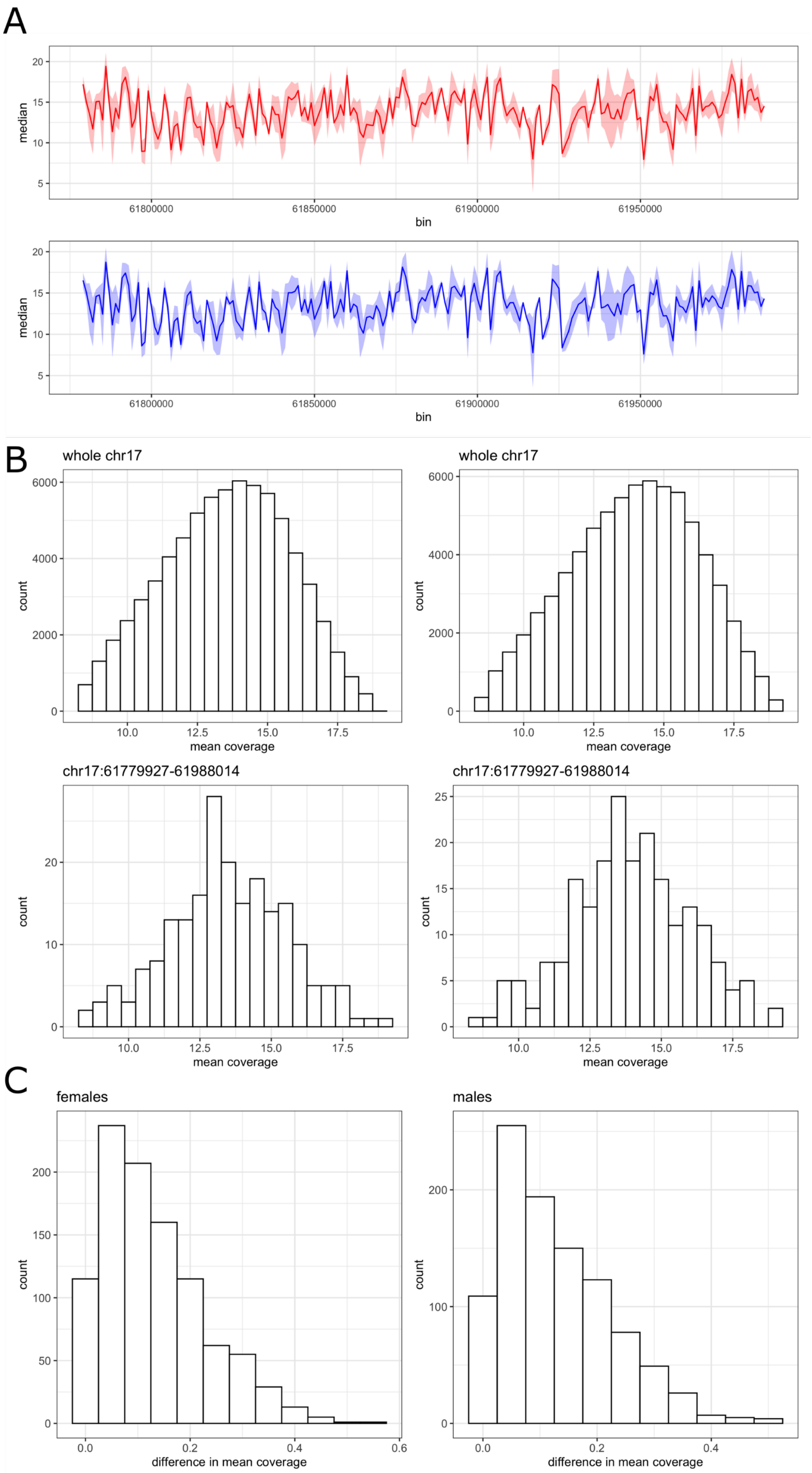

Figure II.2- Coverage analyses for the X chromosome region. A- Mean coverage for males (red) and females (blue), the ribbon corresponds to the mean +- sd, for the region of interest. **B-** Distribution of the mean coverage for females and males for the whole X chromosome without the PARs (top panels) and for the region of interest for females and males (bottom panel). **C-** Distribution of the difference between the mean coverage of the region of interest and the mean coverage of a randomly drawn set of 171 bins in the whole X chromosome, for 1000 random samplings.

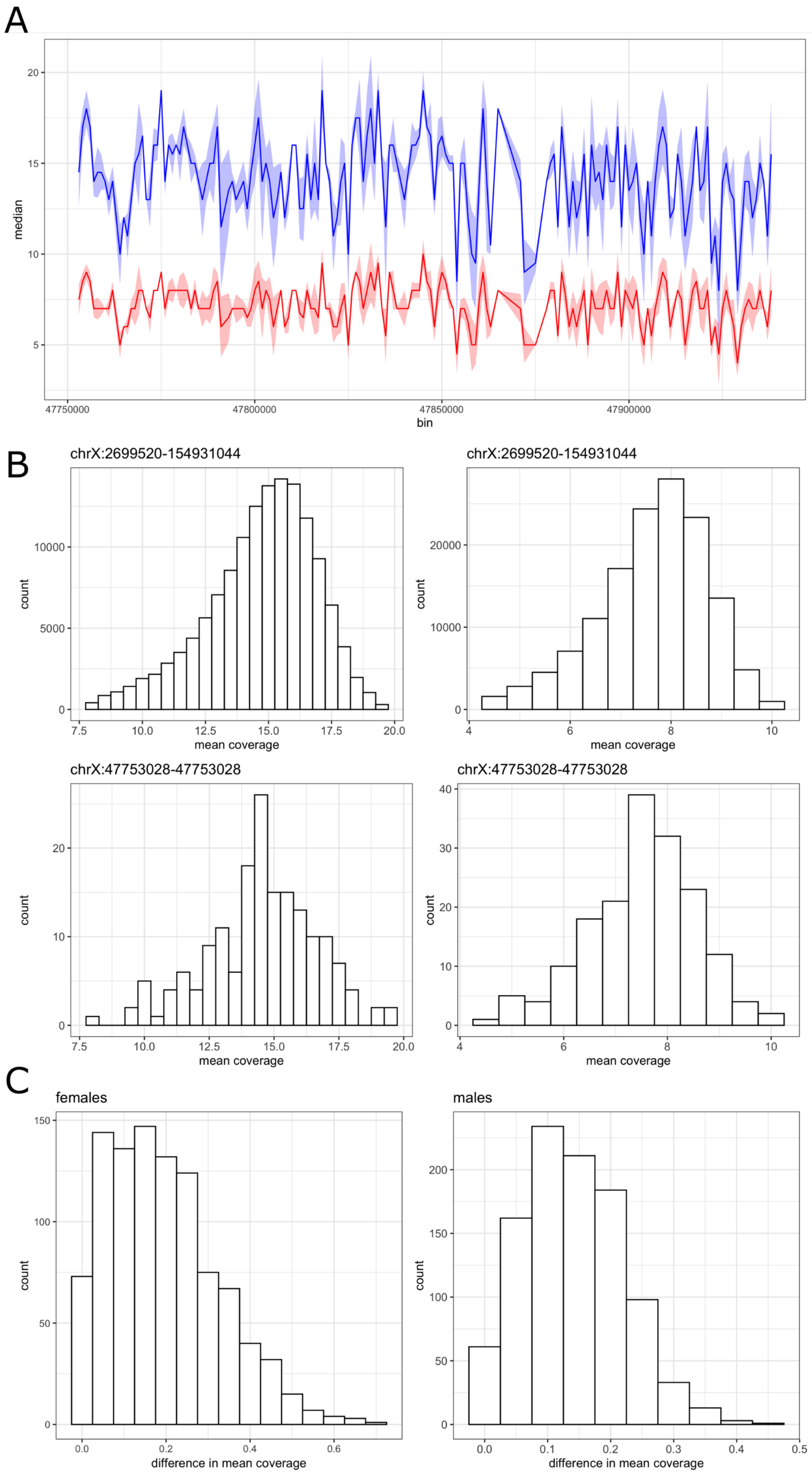

III- **Replication in the 1000Genomes populations**

1. F_{ST} distribution in the candidate regions detected in GoNL

Phase 3 version of the 1000 Genomes dataset was used. We considered European population, CEU (184 Utah resident with Northern and Western European Ancestry), TSI (112 Toscani in Italy), IBS (162 Iberian populations in Spain), GBR (107 British in England and Scotland) and FIN (105 Finnish in Finland). We analyzed the pattern of variation of intersexual F_{ST} within the strongest candidate regions (xi=2) in the European populations from the 1000 Genomes project (Auton et al., 2015). At the population level, we expect the intersexual F_{ST} signal to decrease with distance from the genomic location of a SA locus (Kirkpatrick & Guerrero, 2014). We used the middle of each candidate region detected in GoNL as a proxy for the average location of causal variants, if present. We restricted our analysis to SNPs +/-15kb apart from the middle of the regions, which comprises ~50% of all SNP (Figure III.1). Simple linear regression analysis showed that intersexual F_{ST} decreased with distance in SA regions (beta = -1.81×10^{-4}, *p*-value = 9×10^{-5}), but not in SD regions (beta = -7.37×10^{-5}, *p*-value = 0.15) nor in SL regions (beta = 8.69×10^{-5}, *p*-value = 0.12) (Figure III.2 and Table III.1). For SA regions, the relationship between intersexual F_{ST} and genomic distance was significantly negative in GBR, FIN and IBS while no signal was found in TSI and CEU (Figure III.3 and Table III.2).

Table III.1- Simple regression analysis in all European populations for different candidate regions: slope (beta), *p*-value and R^2

Type	beta	*p*-value	R^2
**Sex Antagonistic**	-1.81E-04	**9.00E-05**	3.77E-03
**Sex Differential**	-7.37E-05	1.51E-01	4.13E-04
**Sex Limited**	8.69E-05	1.20E-01	1.15E-03

Table III.2- Simple regression analysis in SA regions for different populations: slope (beta), *p*-value and R^2

Population	beta	*p*-value	R2
GBR	-4.39E-04	**4.92E-04**	1.45E-02
FIN	-1.87E-04	**1.48E-03**	1.26E-02
IBS	-3.14E-04	**3.22E-03**	1.06E-02
TSI	-6.96E-05	5.19E-01	5.22E-04
CEU	8.18E-05	3.40E-01	1.12E-03

Figure III.1- Distribution of distance from the center of the candidate regions

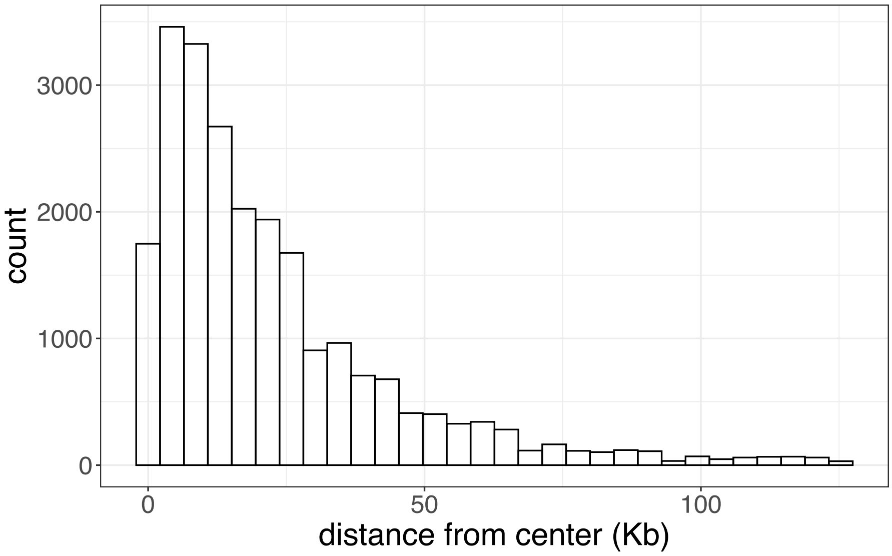

Figure III.2- Intersexual F_{ST} in relation to the distance from the center of the candidate regions in the pooled European populations, for each region type.

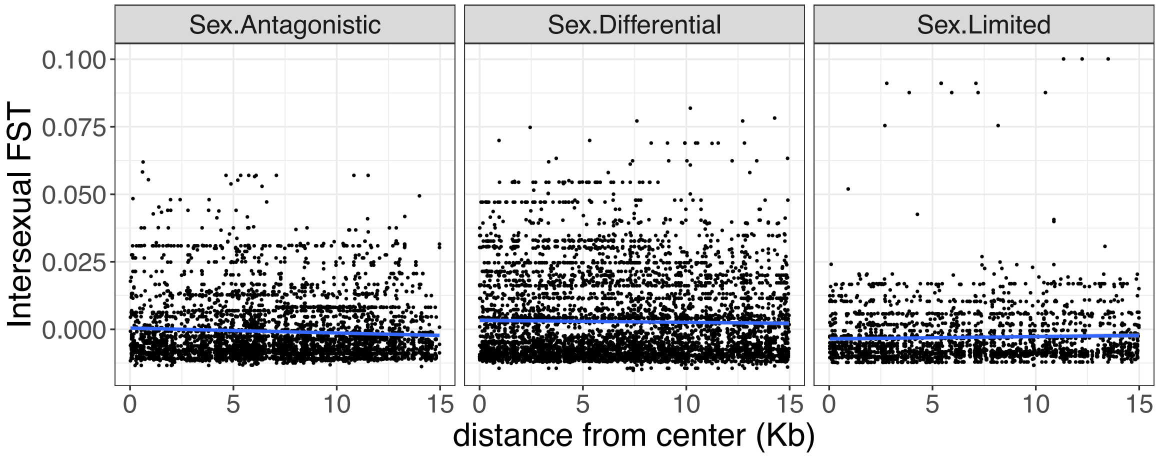

Figure III.3- **Intersexual F_{ST} in relation to the distance from the center of the candidate regions** in each European populations, for sex-antagonistic regions only.

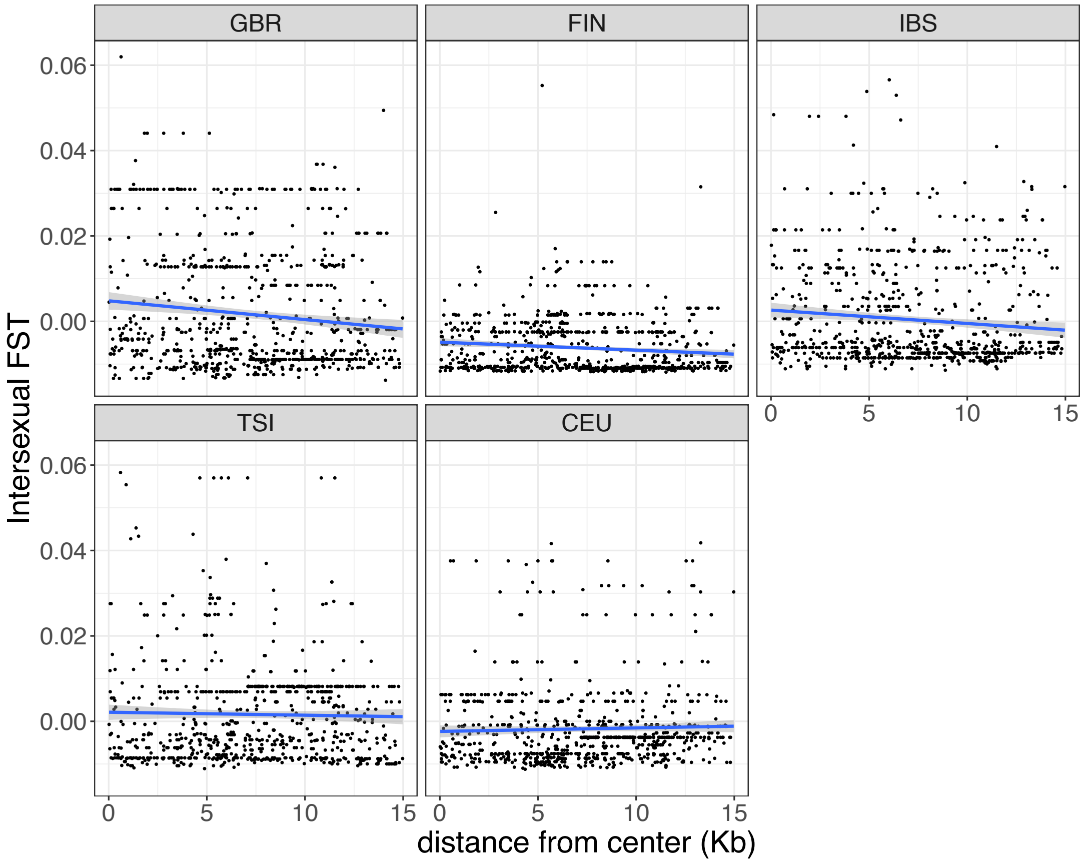

2. Haplotype analyses for the two candidate regions

We extracted the phased genotypes of both candidate regions for all individuals included in the 1000 Genomes Phase 3. Then, we computed the matrix of distances between haplotypes within each populations, and performed a Multi-Dimensional Scaling (MDS). We report here the results for European populations CEU (184 Utah resident with Northern and Western European Ancestry), TSI (112 Toscani in Italy), IBS (162 Iberian populations in Spain), GBR (107 British in England and Scotland) and FIN (105 Finnish in Finland). For the chromosome 17 region, the three haplotypes are present in all European populations in the 1000Genomes dataset (Figure III.3). For the X-linked region, we can see individuals bearing at least 3 out of the 4 haplotypes detected in GoNL (haplotype 1, 2 and 4, Figure III.4). We did not find an excess of males or females in one haplotype for either region (Table III.3 and III.4). Note that the haplotypes are labelled 1, 2, 3 or 0 but that one haplotype might not have the same label (0, 1 ,2 or 3) in different populations.

Table SIII.3- For the European population of 1000 Genomes, counts of males and females for each haplotype of chr17:61779927-61988014, expected proportion under the null hypothesis (males and females are equally distributed), observed proportion and Binomial test *p*-value. Haplotype 0 corresponds to haplotypes that could not be categorized with the other haplotypes.

pop	total nb males	total nb females	haplotype	nb males	nb females	expected proportion	observed proportion	Binom *p*-value
CEU	98	100	1	28	37	0.495	0.431	3.23E-01
			2	42	39		0.519	7.39E-01
			3	27	20		0.574	3.09E-01
			0	1	4		0.200	3.75E-01
TSI	106	108	1	29	24	0.495	0.547	4.94E-01
			2	51	50		0.505	9.21E-01
			3	22	24		0.478	8.83E-01
			0	4	10		0.286	1.80E-01
GBR	92	90	1	22	29	0.505	0.431	3.28E-01
			2	31	26		0.544	5.98E-01
			3	35	31		0.530	7.13E-01
			0	4	4		0.500	1.00E+00
FIN	76	122	1	31	47	0.384	0.397	8.17E-01
			2	18	49		0.269	5.92E-02
			3	21	24		0.467	2.84E-01
			0	6	2		0.750	6.13E-02
IBS	108	106	1	34	44	0.505	0.436	2.58E-01
			2	35	31		0.530	7.13E-01
			3	33	29		0.532	7.04E-01
			0	6	2		0.750	2.89E-01

Table SIII.4- For the European population of 1000 Genomes, counts of males and females for each haplotype of chrX:47753028-47938680, expected proportion under the null hypothesis (males and females are equally distributed), observed proportion and Binomial test *p*-value. Haplotype 0 corresponds to haplotypes that could not be categorized with the other haplotypes.

pop	total nb males	total nb females	haplotype	nb males	nb females	expected proportion	observed proportion	Binom *p*-value
CEU	49	100	1	24	51	0.329	0.320	1.00E+00
			2	22	41		0.349	7.89E-01
			0	3	8		0.273	1.00E+00
TSI	53	108	1	24	63	0.329	0.276	3.07E-01
			2	23	36		0.390	3.34E-01
			0	6	9		0.400	5.87E-01
GBR	46	90	1	21	37	0.338	0.362	6.80E-01
			2	20	51		0.282	3.80E-01
			0	5	2		0.714	4.82E-02
FIN	38	122	1	11	57	0.238	0.162	1.56E-01
			2	22	51		0.301	2.15E-01
			0	5	14		0.263	7.89E-01
IBS	54	106	1	25	62	0.338	0.287	3.65E-01
			2	24	33		0.421	2.07E-01
			0	5	11		0.313	1.00E+00

Figure III.3- Multi Dimensional Scaling of the region of chr17:61779927-61988014 for the European population of 1000 Genomes. CEU (184 Utah resident with Northern and Western European Ancestry), TSI (112 Toscani in Italy), IBS (162 Iberian populations in Spain), GBR (107 British in England and Scotland) and FIN (105 Finnish in Finland).

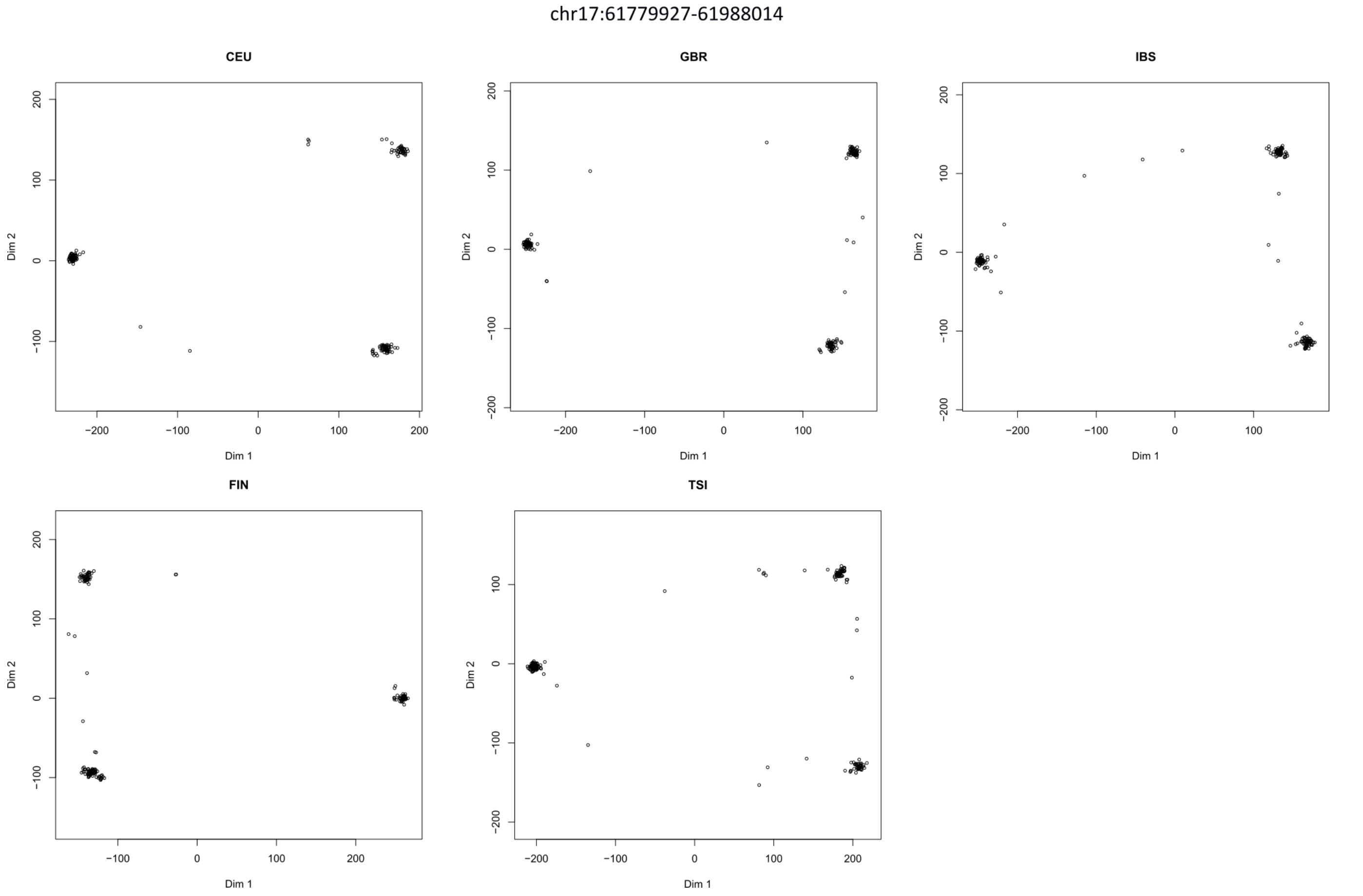

Figure III.4- Multi Dimensional Scaling of the region of chrX:47753028-47938680 for the European population of 1000 Genomes. CEU (184 Utah resident with Northern and Western European Ancestry), TSI (112 Toscani in Italy), IBS (162 Iberian populations in Spain), GBR (107 British in England and Scotland) and FIN (105 Finnish in Finland).

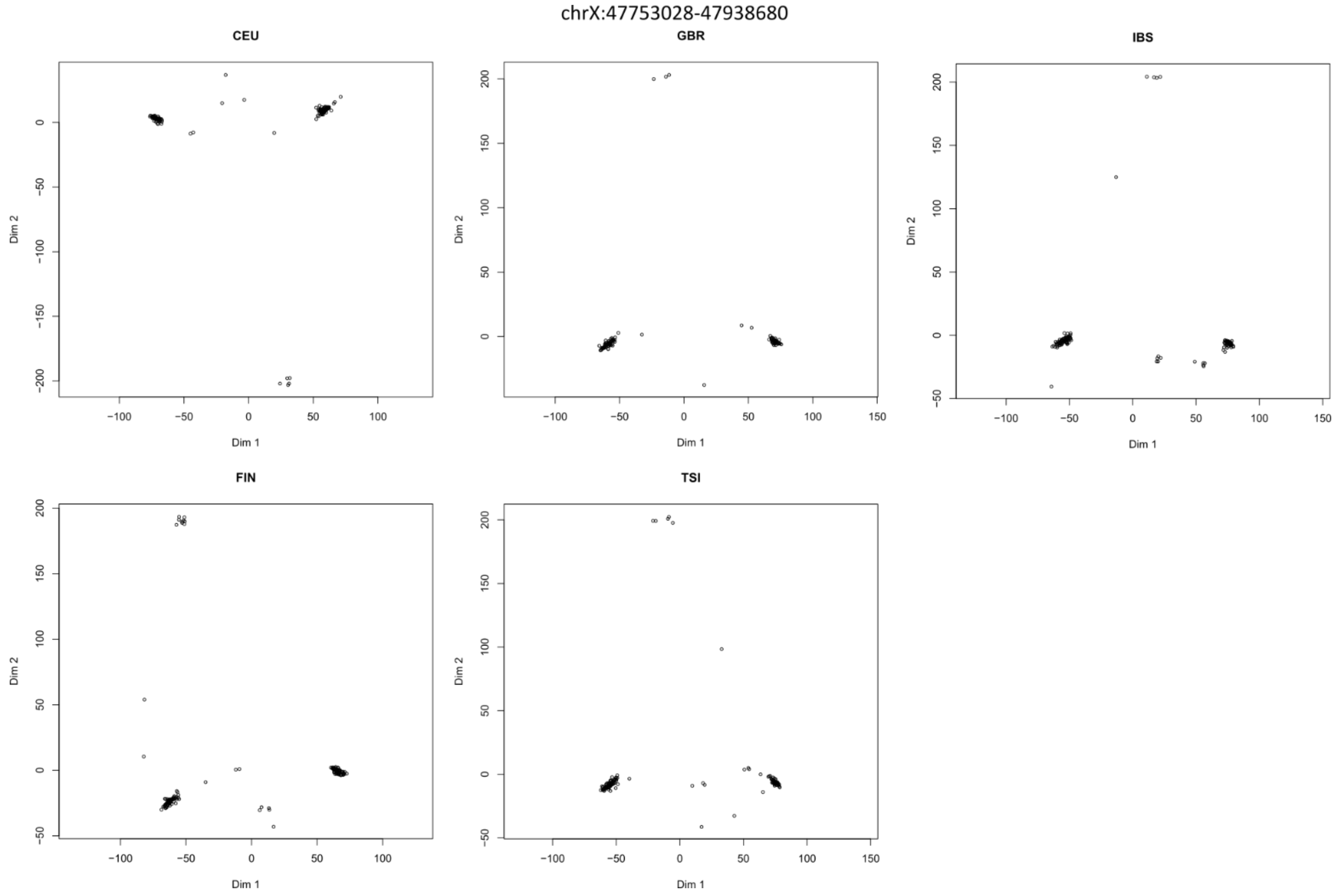

SUPPLEMENTARY FIGURES

Figure S1: Histogram of the thinned *p*-values for 5272 SNPs that are 500kb apart.

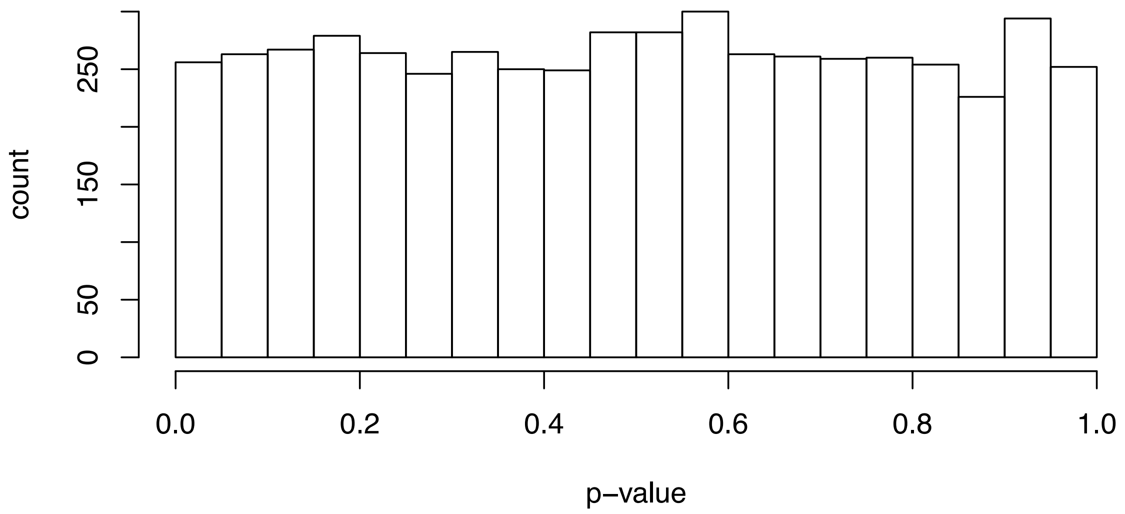

Figure S2 – Intersexual F_{ST} distributions in both parents and offsprings in 248 trios for the three types of TD regions (in red) and for random regions with matching heterozygosity, length and number of SNPs (black).

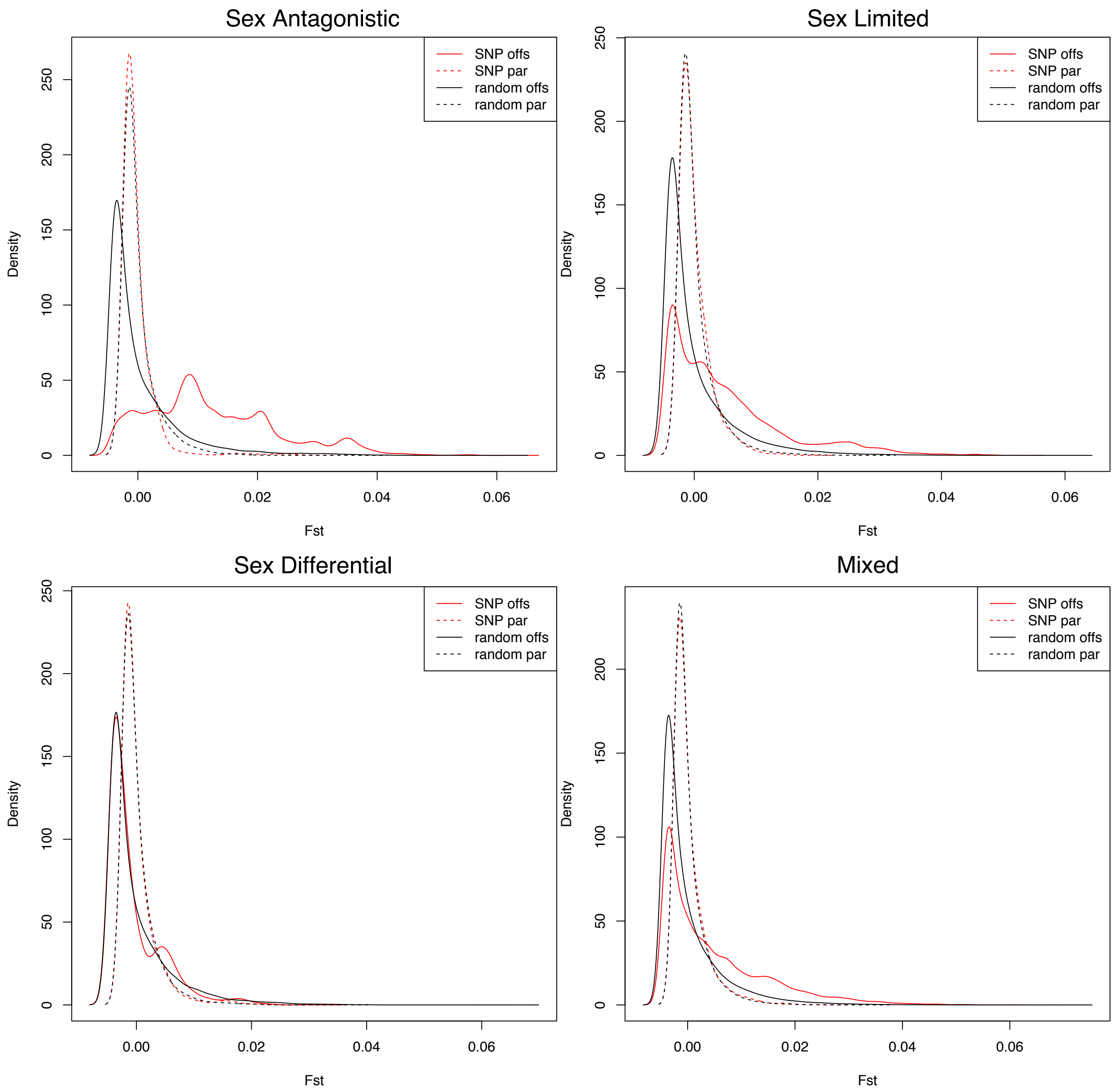

Figure S3- Sexually antagonistic TD candidate regions A,B- chr17:61779927-61988014 (208 kb) **C,D-** chrX:47753028-47938680 (186 kb) **A,C-** intersexual F_{ST} in parents (red) and offspring (blue) for the extended region, significant *p*-values (as calculated by Fisher exact test, *p*-value < 0.05) are in lighter color. **B,D-** The female (red) estimates of recombination rates from our combined genetic maps. Horizontal grey lines represent the position of the region of interest.

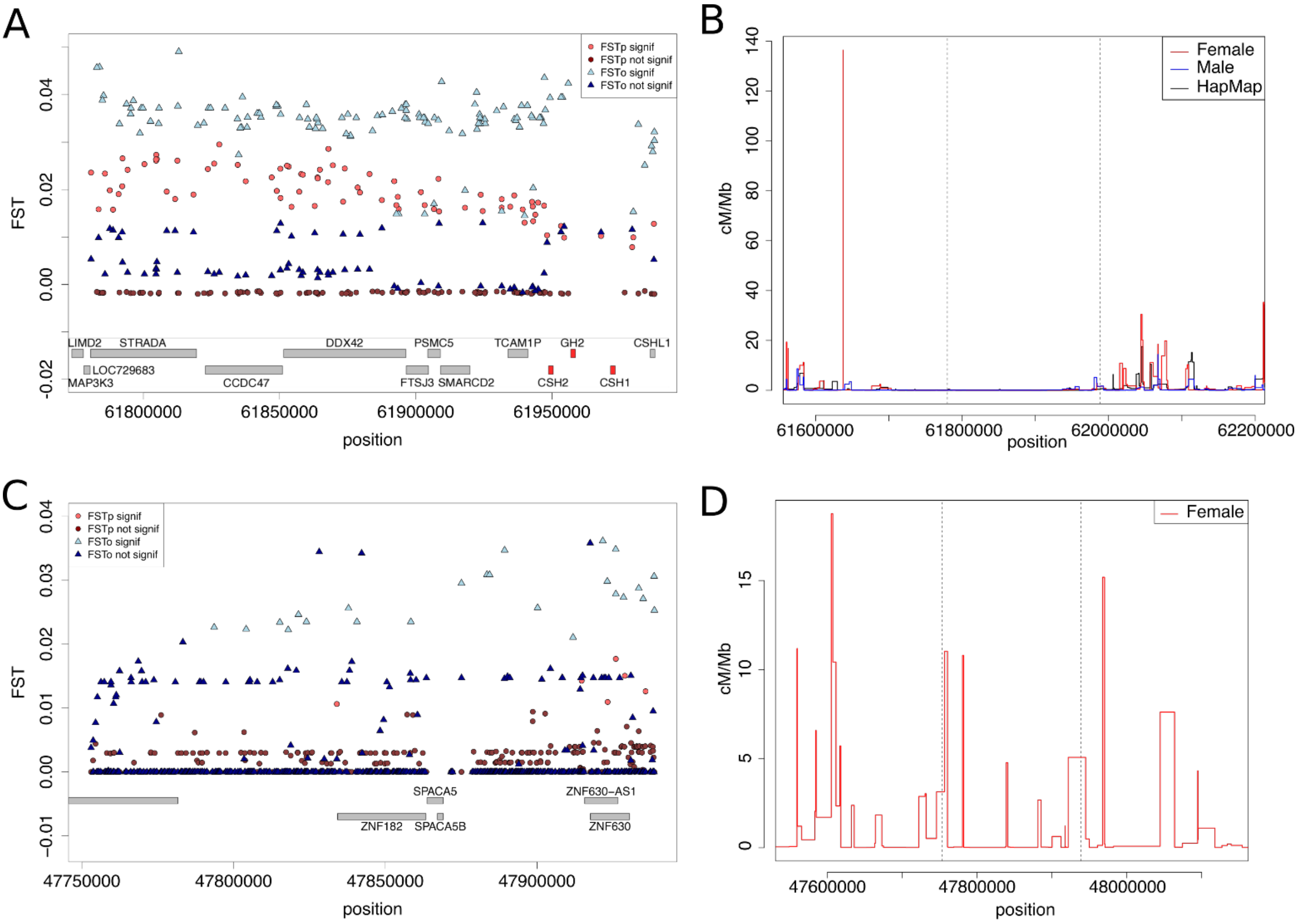

Figure S4- Genetic divergence between the three haplotypes discovered on region chr17:61779927-61988014 (208 kb): each line represents an individual and each column represent a SNP. A SNP is colored in grey if its genotype is the reference allele and in blue if it is the alternative allele.

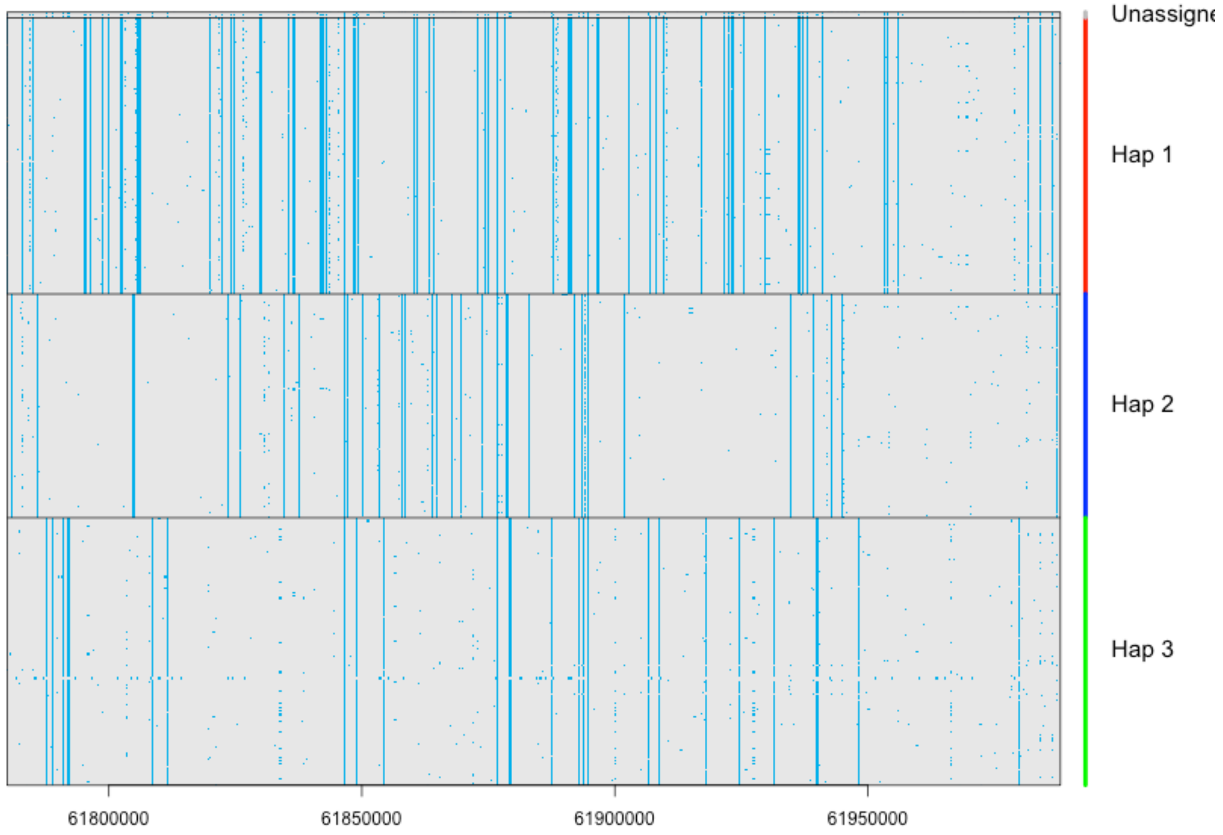

Figure S5- Genetic divergence between the four haplotypes discovered on region chrX:47753028-47938680 (186 kb): each line represents an individual and each column represent a SNP. A SNP is colored in grey if its genotype is the reference allele and in blue if it is the alternative allele.

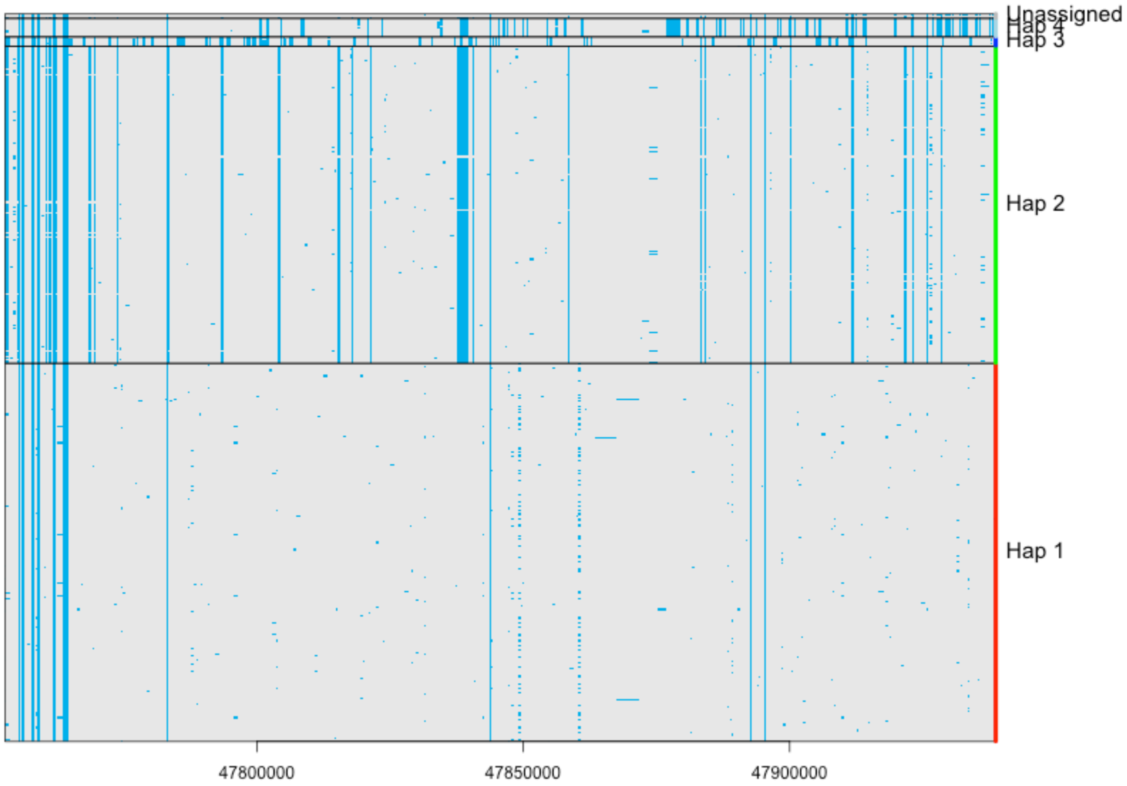

Figure S6- Sexually antagonistic TD candidate regions chr2:229374126-229499707 (125kB), the SA region displaying the highest mean ε_f. **A-** Sex-specific distortion coefficients epsilon (ε_m in red and ε_f in blue). Epsilon differing significantly from zero (LRT *p*-values lower than 0.05) are in lighter colors. **B -** Multi Dimensional Scaling on the genetic distance matrix between all haplotypes (two per individual) based on the phased data, clustered using a density-based clustering algorithm. Each color denotes a cluster. **C -** Genetic divergence between the three haplotypes discovered: each line represents an individual and each column represent a SNP. A SNP is colored in grey if its genotype is the reference allele and in blue if it is the alternative allele.

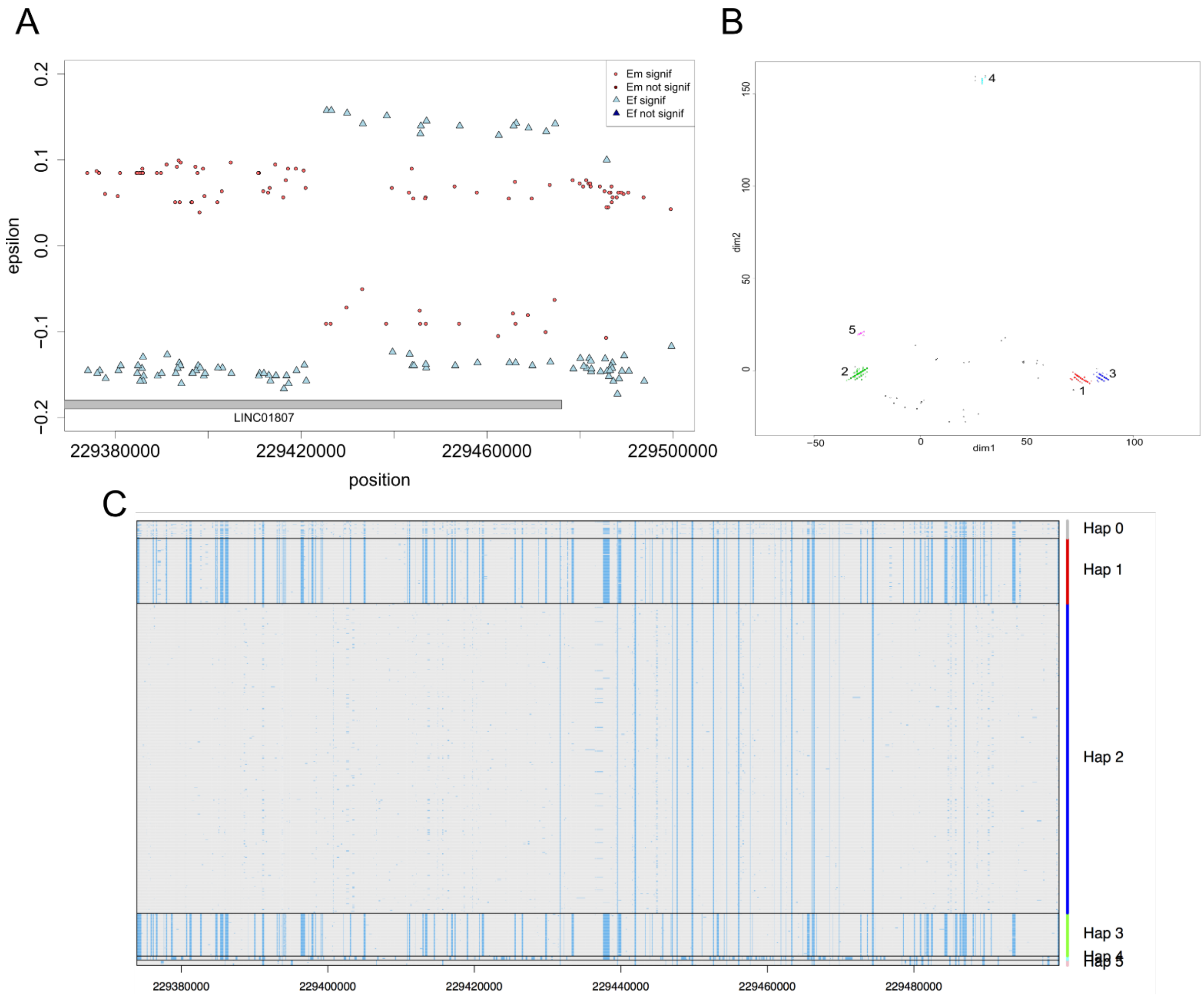

Figure S7- Sexually antagonistic TD candidate regions chr5:168493019-168513628 (20kB), the SA region displaying the highest delta epsilon ($|\varepsilon_m - \varepsilon_f|$) and the highest mean ε_m. **A-** Sex-specific distortion coefficients epsilon (ε_m in red and ε_f in blue). Epsilon differing significantly from zero (LRT *p*-values lower than 0.05) are in lighter colors. **B -** Multi Dimensional Scaling on the genetic distance matrix between all haplotypes (two per individual) based on the phased data, clustered using a density-based clustering algorithm. Each color denotes a cluster. **C -** Genetic divergence between the three haplotypes discovered: each line represents an individual and each column represent a SNP. A SNP is colored in grey if its genotype is the reference allele and in blue if it is the alternative allele.

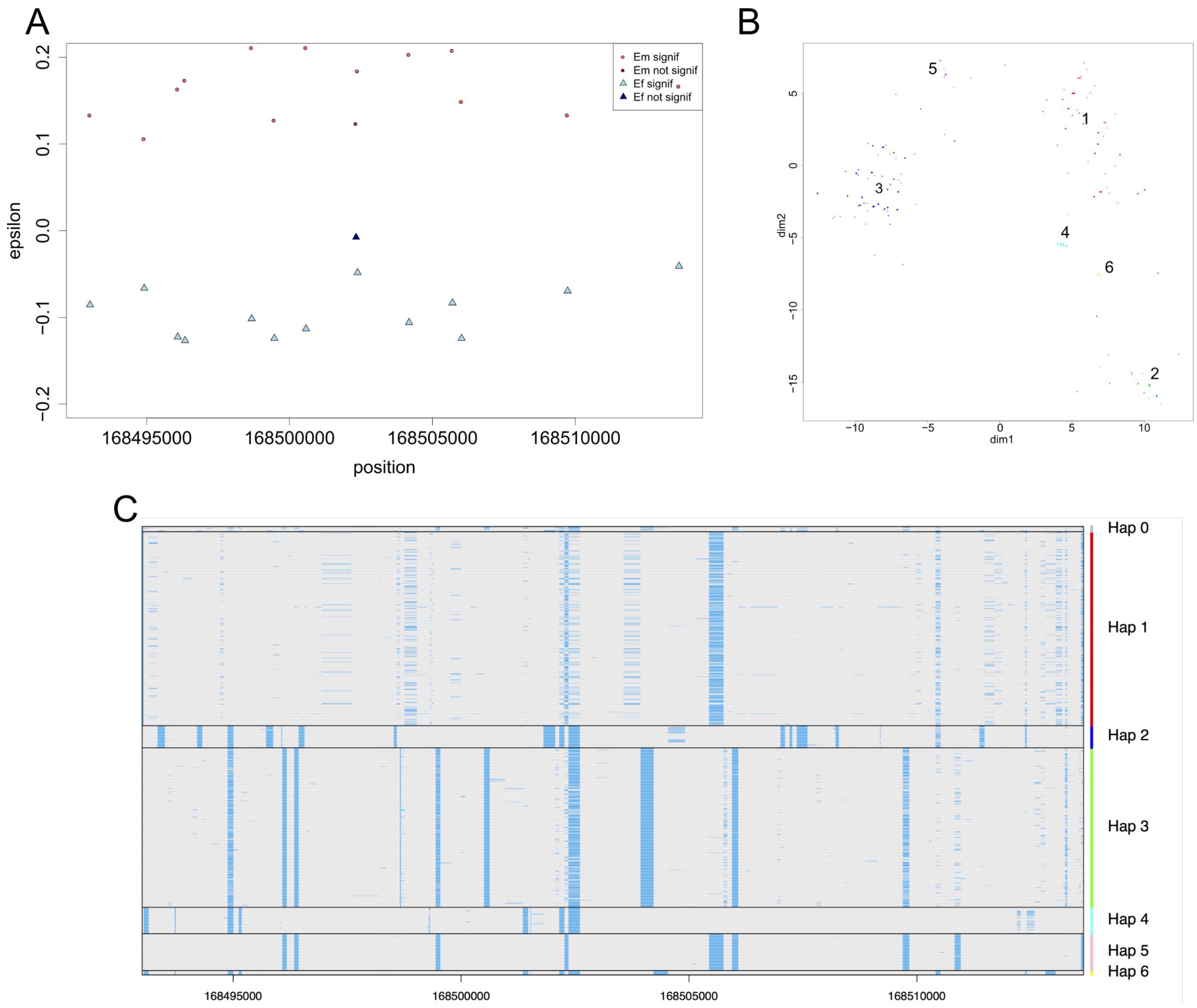

Figure S8- Sexually antagonistic TD candidate regions chr7:65559180-66028495 (469kB), the SA region displaying the lowest delta epsilon ($|\varepsilon_m - \varepsilon_f|$). **A-** Sex-specific distortion coefficients epsilon (ε_m in red and ε_f in blue). Epsilon differing significantly from zero (LRT *p*-values lower than 0.05) are in lighter colors. **B -** Multi Dimensional Scaling on the genetic distance matrix between all haplotypes (two per individual) based on the phased data, clustered using a density-based clustering algorithm. Each color denotes a cluster. **C -** Genetic divergence between the three haplotypes discovered: each line represents an individual and each column represent a SNP. A SNP is colored in grey if its genotype is the reference allele and in blue if it is the alternative allele.

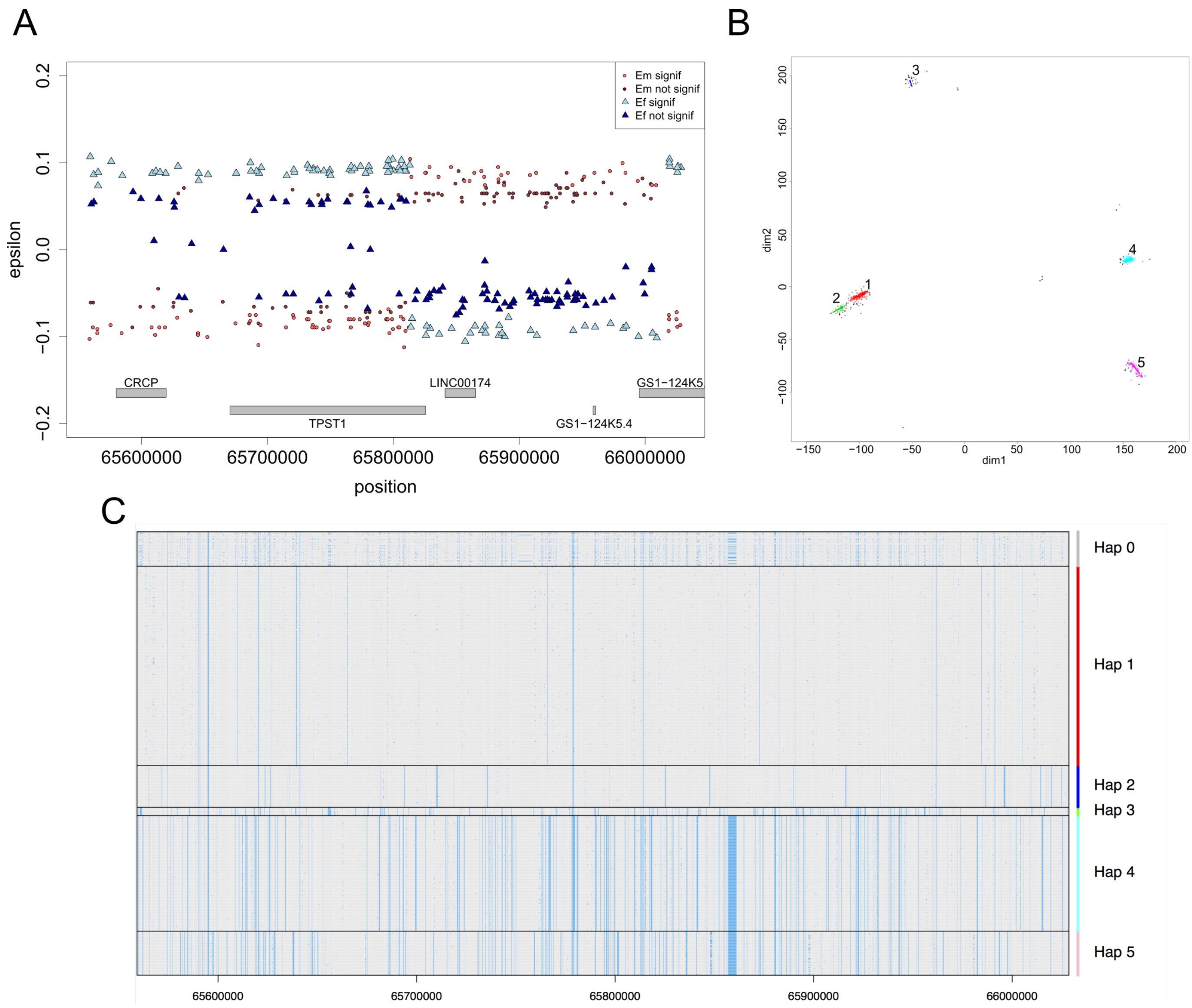

Figure S9- Sexually antagonistic TD candidate regions chr19:15838972-15893942 (55kB), the SA region displaying the lowest mean ε_m. **A-** Sex-specific distortion coefficients epsilon (ε_m in red and ε_f in blue). Epsilon differing significantly from zero (LRT *p*-values lower than 0.05) are in lighter colors. **B -** Multi Dimensional Scaling on the genetic distance matrix between all haplotypes (two per individual) based on the phased data, clustered using a density-based clustering algorithm. Each color denotes a cluster. **C -** Genetic divergence between the three haplotypes discovered: each line represents an individual and each column represent a SNP. A SNP is colored in grey if its genotype is the reference allele and in blue if it is the alternative allele.

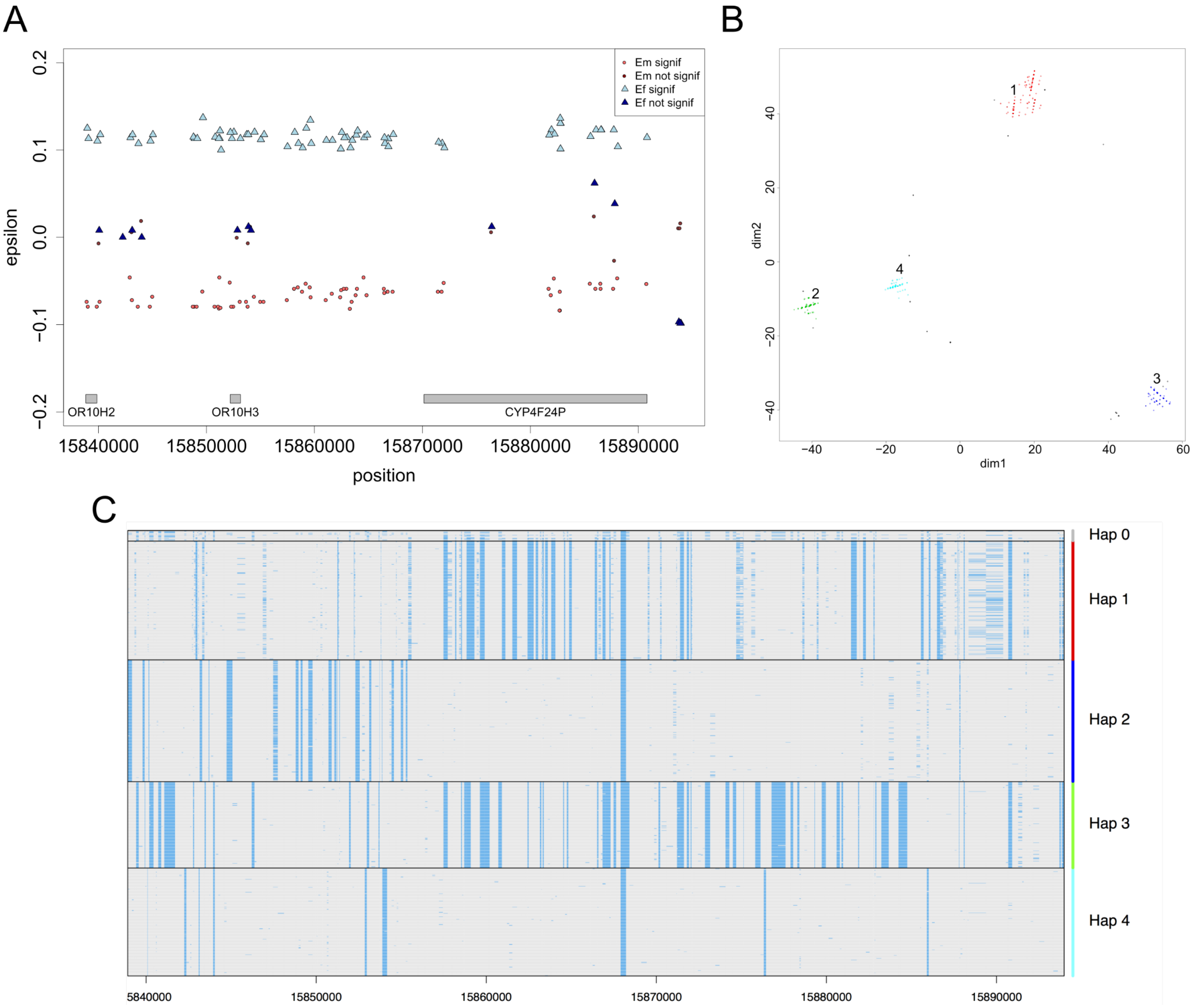

Figure S10- MDS of the genetic distances between all individuals (first two dimensions) for autosomes (1 million randomly drawn SNP) and the X chromosome (random draw of an X chromosome in females). The blue squares are females and the red circles are males.

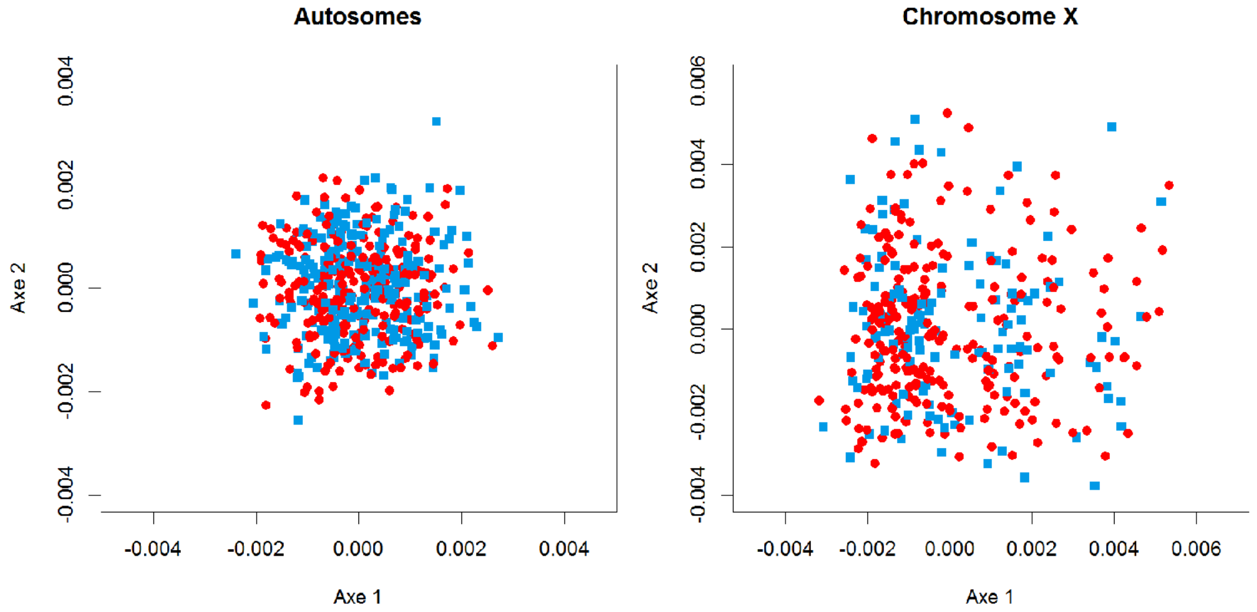

Figure S11- Distribution of the age at sampling for mothers (median=61), fathers (median=63), daughters (median=35) and sons (median=34).

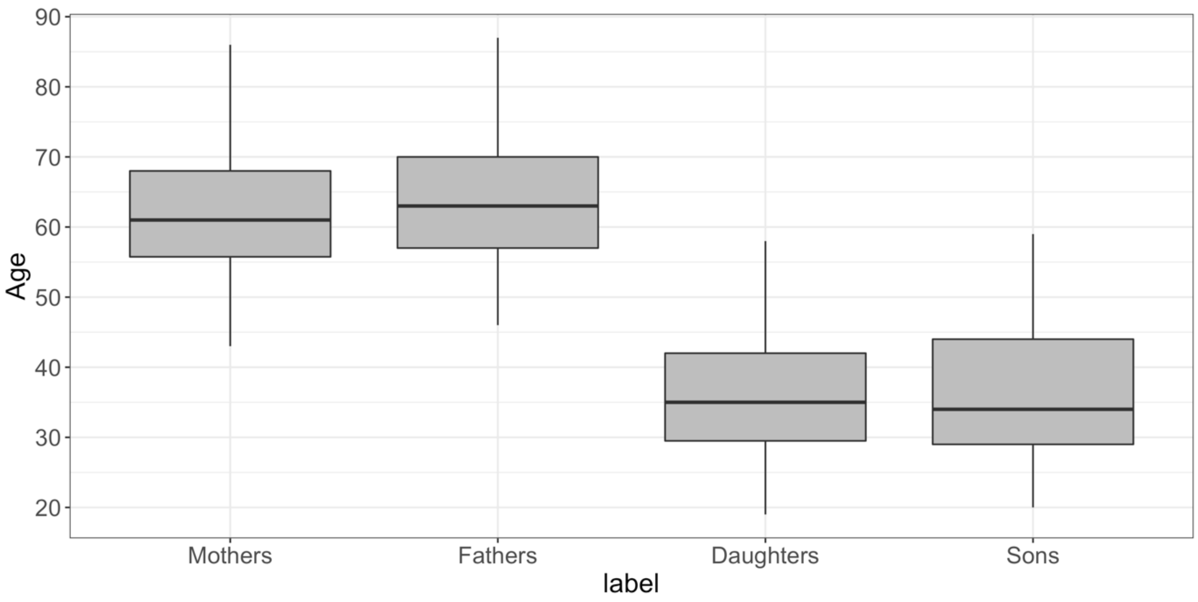

SUPPLEMENTARY TABLES

Table S2 - Summary of the enrichment found for the list of genes present in SD TD regions. For GO Biological Process and Human Gene Atlas, the enrichment is based on biological functions. For both GTEX and Jensen Tissue databases, the enrichment is based on which tissues the genes are expressed in.

Database	Term	Overlap	*p*-value	Adjusted *p*-value	*Z*-score	Combined Score	Genes
**GO Biological Process**	choline catabolic process (GO:0042426)	2/7	2.88E-04	5.14E-02	-2.81	22.91	DMGDH;BHMT
	L-methionine salvage (GO:0071267)	2/8	3.83E-04	5.14E-02	-2.63	20.66	BHMT;BHMT2
	cellular biogenic amine catabolic process (GO:0042402)	2/9	4.91E-04	5.14E-02	-2.87	21.88	DMGDH;BHMT
**Human Gene Atlas**	Optic nerve hypoplasia (HP:0000609)	2/24	3.63E-03	2.22E-01	-2.32	13.02	DDHD2;FGFR1
**GTEX Tissue Expression Profile down**	GTEX-VJYA-0126-SM-4KL1P_spleen_male_60-69_years	6/304	9.81E-04	1.00E+00	-1.88	12.99	DMGDH;BHMT;BHMT2;NDNF;PPL;FGFR1
	GTEX-PW2O-0126-SM-48TC8_spleen_male_20-29_years	6/311	1.10E-03	1.00E+00	-1.82	12.39	DMGDH;BHMT;BHMT2;NDNF;PTCHD4;PPL
	GTEX-POMQ-0126-SM-48TD6_spleen_female_20-29_years	5/341	9.12E-03	1.00E+00	-1.73	8.12	DMGDH;BHMT2;NDNF;PTCHD4;PPL
	GTEX-UPK5-1626-SM-4JBHI_spleen_male_40-49_years	5/353	1.05E-02	1.00E+00	-1.77	8.04	DMGDH;BHMT2;NDNF;BCAT1;PPL
**GTEX Tissue Expression Profile up**	GTEX-T5JW-1426-SM-4DM5Q_esophagus_female_20-29_years	7/621	8.62E-03	1.00E+00	-1.68	7.97	BHMT;BHMT2;LSM1;CCDC102A;LETM2;GLYR1;FGFR1

Table S3- Summary of the enrichment found for the list of genes present in SL TD regions. For GO Biological Process and Human Gene Atlas, the enrichment is based on biological functions. For both GTEX and Jensen Tissue databases, the enrichment is based on which tissues the genes are expressed in.

Database	Term	Overlap	*p*-value	Adjusted *p*-value	*Z*-score	Combined Score	Genes
**GO Biological Process**	tRNA catabolic process (GO:0016078)	2/7	1.52E-03	2.54E-01	-3.11	20.19	EXOSC9;EXOSC8
**Human Gene Atlas**	Goiter (HP:0000853)	3/42	5.70E-03	3.92E-01	-2.16	11.16	TG;EYA1;SDHD
	Recurrent bacterial skin infections (HP:0005406)	2/13	5.45E-03	3.92E-01	-2.05	10.68	NCF2;LYST
**GTEX Tissue Expression Profile down**	GTEX-S3XE-0626-SM-4AD6B_spleen_male_50-59_years	8/308	5.47E-03	1.00E+00	-1.89	9.86	GRIA1;ALDH1L1-AS2;EYA1;ELOVL2;LAMA4;IPP;KCNK2;ULBP3
**GTEX Tissue Expression Profile up**	GTEX-POYW-1226-SM-2XCEP_lung_male_60-69_years	27/2090	2.21E-02	1.00E+00	-1.68	6.41	GRIA1;USP53;SYCP2L;NR3C1;LYST;ORC4;HERC3;CHORDC1;EPC1;GPBP1L1;SUPT20H;HLA-V;UBQLN4;TRPC3;API5;TPK1;IRAK3;ARPC5;FAM13A-AS1;TTC17;WDR11;ADAT2;PHF20L1;RIT1;ARHGEF2;RBMS2;CSNK1A1L

Table S4 – Summary of the enrichment found for the list of genes present in mixed TD regions. For GO Biological Process and Human Gene Atlas, the enrichment is based on biological functions. For both GTEX and Jensen Tissue databases, the enrichment is based on which tissues the genes are expressed in.

Database	Term	Overlap	*p*-value	Adjusted *p*-value	*Z*-score	Combined Score	Genes
**GO Biological Process**	positive regulation of protein localization to cell periphery (GO:1904377)	7/47	4.84E-05	7.99E-02	-1.47	14.57	DPP10;PRKCI;EPB41L2;RANGRF;STX4;EPHA3;PLS1
**GTEX Tissue Expression Profile down**	GTEX-T5JW-0326-SM-4DM6J_fallopian tube_female_20-29_years	7/117	1.11E-02	1.00E+00	-2.03	9.16	ACBD5;PPFIBP2;STX19;DIRAS2;DEPDC1;NEB;SLC12A6
**Mammalian Phenotype**	MP:0011096_embryonic_lethality_between_implantation_and_somite_formation,_complete_penetrance	12/250	6.23E-03	6.62E-01	-3.64	18.51	CDH1;CHEK1;TOP3A;SETD1A;NMT1;KAT8;STX4;PFAS;MET;KPNB1;THAP11;ATR

Table S5- Transmission counts of the three haplotypes detected in the region chr17:61779927-61988014, for heterozygous parents only. For example, haplotype one (186 cases) was transmitted 51 times out of 76 times to sons, and 44 times out of 110 times to daughters (significance assessed with exact Fisher tests).

Parents		Transmission			
haplotype	counts	to sons	p-value	to daughters	p-value
1	186	51 / 76	**$3.84x10^{-3}$**	44 / 110	**$4.48x10^{-2}$**
2	164	21 / 54	$1.34x10^{-1}$	58 / 110	$6.34x10^{-1}$
3	184	27 / 68	$1.14x10^{-1}$	66 / 116	$1.63x10^{-1}$

Table S6- Distance between the three haplotypes detected in the region chr17:61779927-61988014 in terms of mean number of SNPs. In parenthesis is the mean percentage distance between all individuals (total number of SNPs is 1291).

distance	1	2	3
**1**	0		
**2**	167.66 (13.00%)	0	
**3**	151.78 (11.76%)	84.59 (6.55%)	0

Table S7- Transmission counts of the four haplotypes detected in the region chrX:47753028-47938680, for heterozygous parents only. For example, haplotype one (114 cases) was transmitted 18 times out of 45 times to sons, and 44 times out of 69 times to daughters (significance assessed with exact Fisher tests).

Parents		Transmission			
haplotype	counts	to sons	p-value	to daughters	p-value
1	114	18 / 45	2.33E−01	44 / 69	**2.95E−02**
2	111	26 /43	2.22E−01	24 / 68	**2.05E−02**
3	7	1 / 2	1	2 / 3	1
4	8	2 / 4	1	3 / 4	6.25E−01

Table S8- Distance between the four haplotypes detected in the region chrX:47753028-47938680 in terms of mean number of SNPs. In parenthesis is the mean percentage distance between all individuals (total number of SNPs is 575).

distance	1	2	3	4
**1**	0			
**2**	40.61 (7.06%)	0		
**3**	44.13 (7.67%)	41.4 (7.20%)	0	
**4**	103.77 (18.04%)	118.93 (20.68 %)	146.04 (25.40 %)	0

Table S9- Transmission counts of the haplotypes detected in the additional candidate regions, for heterozygous parents only.

chr2:229374126-229499707

**Parents**		**Transmission**			
haplotype	count	to sons	p-value	to daughters	p-value
1	92	19/45	3.71E-01	32/47	**1.86E-02**
2	142	33/63	8.01E-01	28/79	**1.28E-02**
3	70	17/31	7.20E-01	24/39	2.00E-01
4	7	0/3	2.50E-01	2/4	1.00E+00
5	11	5/6	2.19E-01	1/5	3.75E-01

chr5:168493019-168513628

**Parents**		**Transmission**			
haplotype	count	to sons	p-value	to daughters	p-value
1	179	42/67	**4.98E-02**	44/112	**2.93E-02**
2	41	17/24	6.39E-02	8/17	1.00E+00
3	168	20/71	**3.03E-04**	59/97	**4.17E-02**
4	56	8/19	6.48E-01	18/37	1.00E+00
5	63	18/28	1.85E-01	21/35	3.11E-01
6	11	0/1	1.00E+00	4/10	7.54E-01

chr7:65559180-66028495

**Parents**		**Transmission**			
haplotype	count	to sons	p-value	to daughters	p-value
1	133	31/55	4.19E-01	39/78	1.00E+00
2	64	15/28	8.51E-01	15/36	4.05E-01
3	6	2/3	1.00E+00	0/3	2.50E-01
4	104	13/33	2.96E-01	37/71	8.13E-01
5	55	10/23	6.78E-01	19/32	3.77E-01

chr19:15838972-15893942

**Parents**		**Transmission**			
haplotype	count	to sons	p-value	to daughters	p-value
1	166	27/63	3.14E-01	65/103	**1.01E-02**
2	166	37/61	1.24E-01	41/105	**3.13E-02**
3	108	24/47	1.00E+00	31/61	1.00E+00
4	162	29/63	6.15E-01	47/99	6.88E-01

Table S10§ Distance between the haplotypes detected in the additional candidate regions in terms of mean number of SNPs. In parenthesis is the mean percentage distance between all individuals (total number of SNPs per region is 878, 158, 2581, 394 SNPs, respectively).

chr2:229374126-229499707

	1	2	3	4	5
1	6.80 (0.77%)				
2	104.24 (11.87%)	8.80 (1.00%)			
3	22.11 (2.52%)	115.09 (13.11%)	4.41 (0.50%)		
4	167.81 (19.11%)	169.11 (19.26%)	171.06 (19.48%)	5.67 (0.65%)	
5	108.58 (12.37%)	33.89 (3.86%)	119.74 (13.64%)	149.80 (17.06%)	4.11 (0.47%)

chr5:168493019-168513628

	1	2	3	4	5	6
1	6.06 (3.83%)					
2	21.63 (13.69%)	2.36 (1.50%)				
3	16.12 (10.20%)	25.02 (15.83%)	3.79 (2.40%)			
4	14.49 (9.17%)	20.80 (13.16%)	17.11 (10.83%)	2.03 (1.28%)		
5	10.84 (6.86%)	26.48 (16.76%)	10.15 (6.43%)	18.48 (11.69%)	0.21 (0.13%)	
6	15.88 (10.05%)	20.37 (12.89%)	19.59 (12.40%)	5.67 (3.59%)	21.02 (13.31%)	0.13 (0.08%)

chr7:65559180-66028495

	1	2	3	4	5
1	20.81 (0.81%)				
2	61.18 (2.37%)	15.71 (0.61%)			
3	253.08 (9.81%)	275.99 (10.69%)	23.38 (0.91%)		
4	260.26 (10.08%)	281.86 (10.92%)	321.98 (12.48%)	24.42 (0.95%)	
5	287.00 (11.12%)	308.24 (11.94%)	348.02 (13.48%)	169.64 (6.57%)	23.54 (0.91%)

chr19:15838972-15893942

	1	2	3	4
1	17.79 (4.52%)			
2	84.42 (21.43%)	3.67 (0.93%)		
3	90.07 (22.86%)	98.19 (24.92%)	2.45 (0.62%)	
4	67.21 (17.06%)	36.14 (9.17%)	79.28 (20.12%)	1.69 (0.43%)
